# A Simple Approach for Operando Interface Probing for Batteries: Combining Scanning APXPS with Spectroscopic Recognition

**DOI:** 10.1021/acsami.5c25272

**Published:** 2026-03-18

**Authors:** Qianhui Liu, Laura King, Helena Wagner, Alenka Križan, Laurin Derr, Katie L. Browning, Gabriel M. Veith, Tove Ericson, Robert Temperton, Maria Hahlin

**Affiliations:** † Department of Chemistry-Ångström Laboratory, 8097 Uppsala University, SE-75120 Uppsala, Sweden; ‡ Department of Physics and Astronomy, Uppsala University, SE-75120 Uppsala, Sweden; § National Institute of Chemistry, Hajdrihova 19, 1000 Ljubljana, Slovenia; ∥ Institute of Physical Chemistry, Karlsruhe Institute of Technology, 76131 Karlsruhe, Germany; ⊥ Chemical Sciences Division, 150232 Oak Ridge National Laboratory, Oak Ridge, Tennessee 37831, United States; # MAX IV Laboratory, 6146 Lund University, 225 94 Lund, Sweden

**Keywords:** ambient pressure X-ray photoelectron spectroscopy, solid/liquid interface, Li-ion batteries, operando, data processing, spectro-microscopic imaging, dip-and-pull

## Abstract

Probing the solid/liquid interface of batteries operando/in situ with ambient pressure X-ray photoelectron spectroscopy (APXPS) using the dip-and-pull method remains a challenging endeavor due to spatial and temporal variations in liquid layer shape, thickness, and composition. Monitoring the electrochemical and topographical nature of the liquid edge where the interface is accessed is essential to correctly interpret interfacial spectra. In this work, a methodology combining experimental design and software-based data processing for interface probing is reported. This experimental methodology utilizes continuous motion during fixed-mode APXPS measurements by periodically scanning across the dry electrode and thick electrolyte regions to capture the transitional interface. Two software-based approaches for retrieving the interface spectra are evaluated. In an analysis of the intensity attenuation pattern of a unique electrode signal, interface spectra are recognized at the edge of the intensity transition from electrode to electrolyte. The second method utilizes peak positions for interface identification. Selected spectra with the same peak energies also exhibit the same chemical features, indicating the close correlations between the interface energetics and local chemical compositions. Further, topographical information can be extracted using scanning APXPS by translating spectral intensities into liquid thickness, creating a spectro-microscopic 3D image of the liquid edge region. In the examined systems, the thickness of a propylene carbonate electrolyte edge on both lithium cobalt oxide and gold WE surfaces exhibits a step-jump transition from the thin to thick liquid region. The liquid distribution is also shown to depend on the morphological and chemical nature of the electrode. The imaging provides a better understanding of the relationship between liquid distribution and probed interface features while validating the functionality of the setup.

## Graphical abstract

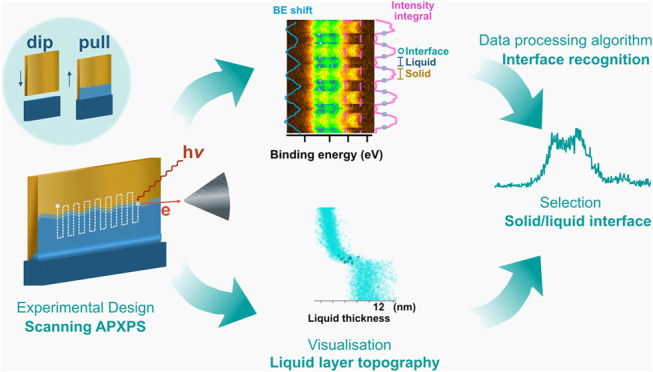

## 1 Introduction

The interfacial nature of electrochemical phenomena such as double layer charging, charge transfer reactions, and solid interphase layer formation has sparked interest in the development of in situ/operando characterization tools capable of probing the solid/liquid interface.^1–3^ Very few techniques are able to directly probe these interfacial processes operando due to instrumentation limitations such as poor chemical sensitivity or inability to measure under local gas/liquid environment change, as seen in scanning tunneling microscopy (STM)^4^ and low energy electron microscopy (LEEM), or a lack of interface/surface sensitivity such as for X-ray diffraction (XRD).^5^ The necessity for operando interface probing capabilities has spurred a wave of methodology developments in many existing analytical fields. For example, in the field of vibrational spectroscopy, both surface and spatial sensitivity has been improved through the development of operando shell-isolated nanoparticle-enhanced Raman spectroscopy (SHINERS/SERS)^6–8^ and operando infrared spectroscopy.^9–13^ Additionally, photoemission electron microscopy (PEEM),^14^ scanning transmission electron microscopy (STEM),^15^ electron energy-loss spectro-imaging (EELS),^16^ X-ray absorption spectroscopy (XAS)^17^ and neutron reflectometry^18,19^ have all been adapted and applied in an operando fashion to further characterize electrified solid–liquid interfaces.

One characterization technique prominently employed in surface/interface science is conventional X-ray photoelectron spectroscopy (XPS).^20,21^ The short inelastic mean free path (IMFP) of the emitted photoelectron allows for excellent surface sensitivity on the scale of several nanometers.^22^ Unfortunately, this property has historically fundamentally limited the measurement to ultrahigh vacuum (UHV) conditions to avoid inelastic scattering of photoelectrons.

More recently, instrumental developments,^23,24^ primarily the introduction of differential pumping systems, has allowed for “near-ambient pressure” conditions in the analysis chamber, enabling the probing of liquid and gas phases. Further developments of ambient pressure XPS involving the introduction of high brilliance synchrotron radiation, combined with chamber designs^25–29^ have enabled operational pressures up to tens of mbar,^30,31^ meaning many liquids with a large range of vapor pressures can now be studied.

However, an experimental challenge for probing the “buried” solid/liquid interface is that one layer (either the solid or liquid phase) needs to be thinner than the probing depth of the photoelectrons. Experimental setups have been designed to access this interface through both the solid phase and the liquid phase. The “liquid-cell” design probes the interface through a nanometer-thick window (e.g., a graphene membrane) which constrains the liquid within a closed cell and acts as the working electrode (WE).^32–36^ Alternatively, a dip-and-pull method can be used to access the interface through a thin layer of liquid that is retained on the electrode surface when the electrodes are partially retracted from a liquid reservoir.^1,25,37–40^

Using a three-electrode system in the dip-and-pull geometry it is possible to simultaneously perform electrochemical experiments while probing the interface region in an operando fashion, as pictured in Figure 1.^41,42^ In the case where charge transfer reactions are ongoing with large currents, the electrolyte layer (some works refer to this as the meniscus) should be thick enough to ensure sufficient ion mass transport and reduce significant *iR* drop relative to the portion of the electrode fully submerged in the bulk electrolyte, as recently explored by Križan et al.^43^ It is thus a delicate balance to form an electrochemically functional electrolyte layer with an interface at an accessible probing depth.^44^

**Figure 1:**
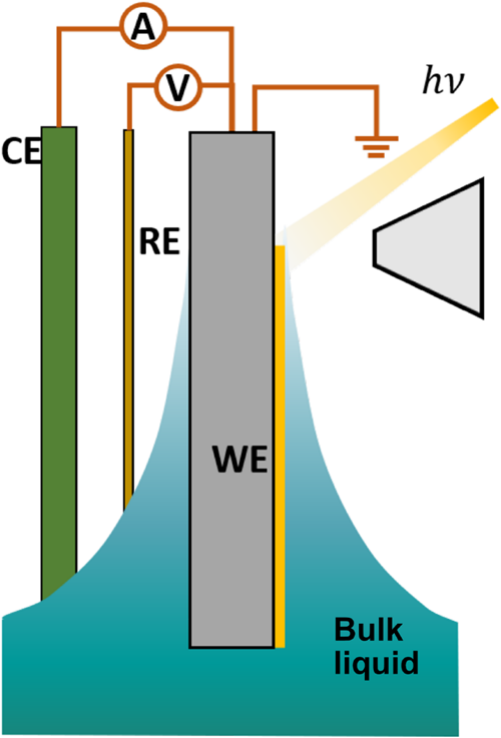
A schematic illustration of the operando electrochemical setup probing the near-liquid edge using the dip-and-pull method. By moving the sample vertically during the XPS measurement, it is possible to probe the interface region near the top edge of the liquid layer. In this study, two systems were examined, Au WE and LCO WE, both in 1 M LiClO_4_/PC electrolyte.

In soft X-ray APXPS (1800 eV used in this study), where an enhanced surface/interface sensitivity is achieved, the probing depth is very much limited. The measurement position for the solid/liquid interface is often located near the top edge of the liquid layer, where XPS signals from both the electrode and electrolyte can be obtained.^45^ This implies that the interface is being probed and is referred to in this article as the “interface region” (Figure 1). The shape and spatial distribution of the liquid layer in the dip-and-pull geometry determine where and how the interface can be accessed. Previously, the liquid layer has been assumed to be a continuous meniscus shape.^1,45,46^ A few studies also mentioned the extension of liquid up the electrode surface that may alter the meniscus shape.^41^ However, the actual shape of the liquid edge, its impact on the interface probing position, and whether the probed region corresponds to a reacted (or dipped) area remain unexplored.

Probing the interface region is further complicated by the nonstatic liquid electrolyte which can be affected by the beam, the electrode surface, and time, as demonstrated within this work.^47^ We therefore present a methodology for solid/liquid interface probing which accommodates the nonuniform and dynamic distribution of liquid at the edge. It can be applied to a range of electrode and electrolyte systems in the dip-and-pull configuration to capture a variety of interfacial processes. The method involves simultaneously spatially scanning in a systematic fashion across the liquid edge while collecting spectra from the interface region, and the dry solid and thick electrolyte regions in close proximity to the liquid edge. This results in the need for a data preprocessing step in order to separate out the interface-specific spectra.

A combination of experimental design and data preprocessing is sometimes used in spectroscopy of dynamic processes to resolve intermediate or minority compounds, however, it is less common in APXPS. One way such a combination is achieved is to induce periodic chemical change in situ by, e.g, alternating potentials^43^ or inducing local changes in gas composition^30,48,49^ in order to capture the onset of any spectral changes. These types of experiments are usually followed by statistical data processing, e.g., automated curve fitting, smoothing, and phase sensitive detection,^50^ or other software-based advanced processing methods, e.g., event-averaging^30^ or advanced Fourier analysis.^48^ In this work, by combining a spatially scanning APXPS experimental approach with a software-based spectroscopic recognition method of data processing, spectra from the interface region can be gathered with better accuracy, good statistics and with negligible influence from irradiation or temporal/spatial variation. The methodology is demonstrated using two systems; a thin film Au WE and a thin film LCO WE, a common Li-ion battery cathode material. Both systems use a 1 M LiClO_4_ PC electrolyte.

This approach is both powerful and feasible with regard to accessing and identifying reliable and consistent solid/liquid interface spectroscopic features when probing nonuniform, dynamic liquid layers while overcoming the limitation of a low S/N ratio. The method requires minimal computational power as it either avoids the need for curve fitting of a large data set or involves relatively simple analysis. The results enable an overview of near-interface chemistry and energetics in the dip-and-pull geometry. This in turn provides a deeper understanding of the formation and distribution of interphase species and of charge transfer mechanisms, as well as their influence on electrode and electrolyte chemistry. The methodology also enables spectro-microscopic imaging of the liquid edge, visualizing the chemical distribution and further, the liquid topography on the electrode surface, which assists in examining the functionality of the dip-and-pull setup in different electrode/electrolyte systems.

## 2 Results

### 2.1 Scanning APXPS Measurements at the Near-Interface Region

Performing APXPS measurements in the dip-and-pull setup faces a number of challenges relating to temporal and spatial variation in the distribution of the liquid layer which can arise during these experiments. These challenges demonstrated below motivate the development of a new, reliable and repeatable measurement procedure.

Through studying a propylene carbonate (PC) based electrolyte it was found that under PC vapor atmosphere, irradiating the same spot on a lithium cobalt oxide (LCO) electrode led to accumulation of liquid seen as small droplets in Figures 2a and S1. The obtained spectra showed that the electrode signal decayed on the same time scale as the spectral acquisition. The low signal-to-noise (S/N) ratios of the spectra require multiple scans in order to obtain satisfactory statistics for spectral analysis. However, by the 10th scan (approximately 1 min), no Co 2p spectral feature existed (Figures 2b and S23). One way to mitigate this is to conduct the APXPS measurement in a scanning fashion (scanning APXPS) where the measurement position is constantly changed to avoid liquid accumulation while gathering enough statistics to produce interface-featured spectra with good S/N ratios for chemical analysis. However, upon scanning linearly across the top edge of the liquid, the signal intensity of the electrode material can sometimes display irregular variation indicating an uneven, nonlinear, and dynamic liquid edge. This makes it impossible to collect spectra from only the interface region by simple horizontal motion (Figure 2c). Instead it results in spectra from a combination of the dry solid, interface and thick electrolyte regions, as can be seen in (Figure 2d). To account for this, the vertical measurement position must be constantly adjusted to relocate the interface region, making the technique time-consuming, less reproducible, unreliable and difficult to interpret.^45^ Simply summing up the spectra would give misleading information about the interface.^51^ This spatial variation of the liquid edge position is possibly an effect of the balance between capillary forces and gravity, liquid evaporation, and sample morphology. For example, a composite/porous electrode can further make the dry/wet boundary less defined due to capillary forces in microcracks on the surface, resulting in a “wetted” region of the electrode which was not initially dipped. The temporal and spatial variation of the liquid layer in terms of its shape, thickness and composition thus brings challenges for real-time probing of the electrode/electrolyte interface with good S/N ratio for quantitative analysis of chemical compositions and energetics.

**Figure 2:**
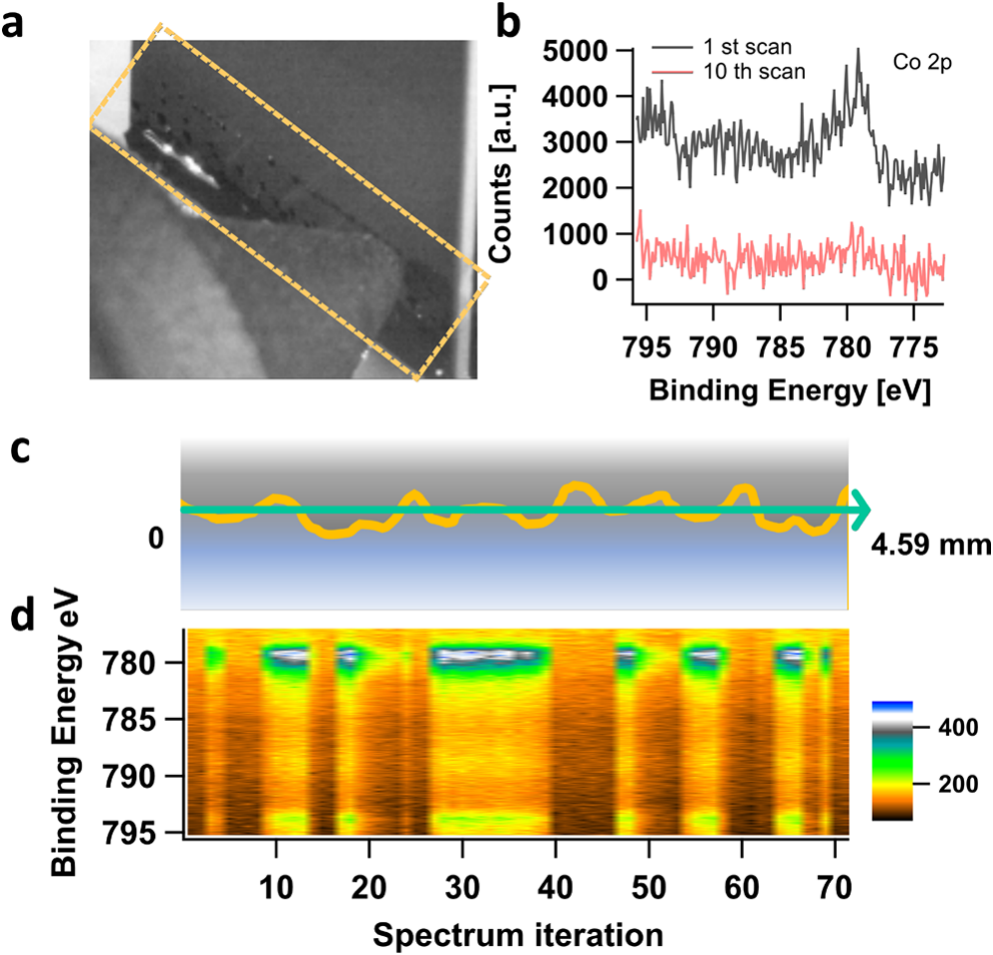
(a) A photo of droplets forming on the surface of the sample at the measurement position, HIPPIE beamline, MAXIV. (b) Co 2p spectra obtained when measuring at a fixed spot at the interface between a LCO thin film electrode and LiClO_4_/PC electrolyte for several minutes. A rapid decay of Co signal is observed within time scale of acquisition. After 10 scans, the spectral feature is barely visible, caused by the accumulation of liquid on the measurement spot. (c) A schematic illustration of the front view of the near-edge liquid distribution. The surface inhomogeneity of the WE surface as well as the effect of gravity and capillary force possibly causing the top-edge of the liquid to be nonhorizontal. (d) A 2D heatmap of Co 2p spectra recorded with a horizontal scanning along the interface over a distance of 4.59 mm. The intensity of the image is plotted vs the binding energy (BE) and the spectrum iteration.

In order to systematically capture the solid/liquid interface in the dip-and-pull setup, a scanning APXPS methodology was employed. A typical three-electrode operando electrochemical setup was used to conduct a potential control experiment while simultaneously recording APXPS measurements whereby the WE was held at an applied voltage and was partially pulled up, exposing a thin liquid layer^42^ (see Section 5). At the top edge of the liquid layer there is a transition from the thick liquid (through which photoelectrons from the solid phase can no longer escape) to undipped solid region. Fixed mode APXPS measurements were subsequently performed while scanning the liquid edge along a programmed square-wave trajectory at a velocity of 0.01–0.02 mm/s in the vertical direction and 0.1 mm/s laterally, covering the liquid edge region spanning 2 mm (vertical) × 6 mm (lateral). The motion and thus measurement position was controlled by synchronous manipulator control of the dipped electrodes and the electrolyte beaker. The theoretical dwell time per spectrum in fixed mode was set to 0.5 s for most examined core levels, and 2.6 s for Co 2p to ensure sufficient S/N ratios. In reality, additional dead time during core-level changes was required for the analyzer voltage to ramp between the different kinetic energies. Therefore, the choice of the manipulator velocity is a compromise between minimizing the irradiation effect and maximizing the spatial resolution given a beam size of 50 × 50 μm. As shown in Figure 3a, all regions of the dry solid and thick liquid in the vicinity of the electrolyte edge are probed. The measurement position moves from undipped solid, to the interface region, to the bulk liquid and back up again while recording XPS spectra in fixed mode. The probed core level is expected to show regular variation in spectral features with regard to the chemical composition and energetics in each region.

**Figure 3:**
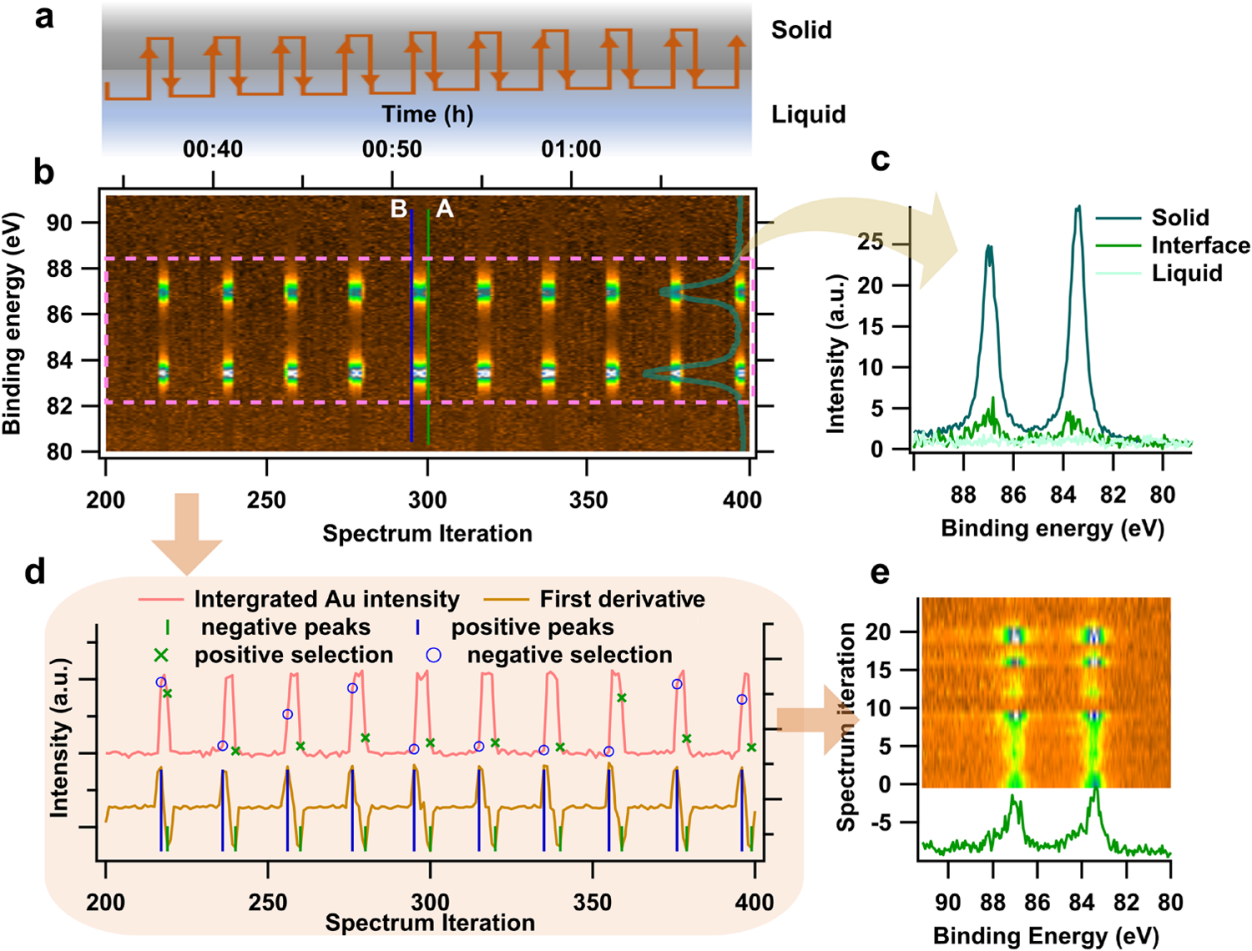
(a) Schematic illustration of scanning APXPS measurements with square-wave path oscillating between dry solid and thick liquid across the interface. (b) The heatmap of 200 out of 500 Au 4f spectra measured with scanning APXPS in a Au WE system with LiClO_4_/PC electrolyte. The image is plotted with *y*-axis as binding energy, *x*-axis bottom as iteration scans, *x*-axis top as time scale, and *z*-axis the intensity showing as the signals of the image. The intensity of the spectra were calibrated by aligning the background level (see notes in SI). (c) Three spectra extracted from panel (b) which are probed consecutively along the vertical direction near the liquid edge (the green line in panel (b) marks the location of spectrum from the solid/liquid interface). As scanning goes from dry solid to thick liquid region, the intensity of Au 4f spectra drops abruptly with no Au 4f feature present in the thick liquid region due to the inelastic scattering of the overlayer. (d) The integrated intensity of the Au signal in the energy range of 82–84 eV (pink dash box region from panel a) vs the spectrum iterations (pink curve), the first derivative of the integrated intensity curve (brown curve), and the searched negative peak position from the first derivative (green sticks). The green/blue mark on the integrated intensity curve shows the intensity of the selected interface spectrum. (e) The heatmap of selected interface-featured spectra, with extracted one spectrum shown in green.

This methodology helps to (1) avoid prolonged beam irradiation on the same spot, (2) monitor changes in spectral features when routinely probing across the dry/wet boundary, (3) provide signal contrast between the solid, interface, and liquid regions for interfacial spectral recognition, and (4) create “repeating” events to improve S/N ratio from the short acquisition time of each spectrum.

Between the two materials examined, Au and thin film LCO, the metallic Au provides a relatively flat surface for the liquid layer to form on, minimizing spatial inhomogeneity, while the porosity of the LCO electrode surface benefits the electrolyte transport with capillary force, but results in a less defined liquid edge between the dipped/undipped regions (see scanning electron microscopy of sample surface in Figure S2). To demonstrate the technique, the scanning APXPS results of the Au system are displayed in detail in Figure 3. Under an applied potential of 0.8 V (vs Li^+^/Li) the Au 4f (Figure 3), O 1s (Figure 4), and C 1s (Figure S3) core levels are consecutively measured. Simultaneously, manipulators of both electrodes and the liquid reservoir are programmed to move in a square-wave motion probing across the liquid edge as demonstrated in Figure 3a. A heat map of the acquired Au 4f spectra is presented in Figure 3b, in which the signals shown at 87 and 83.5 eV correspond to the spin–orbit coupled Au 4f_5/2_ and 4f_7/2_ peaks, respectively. As the measurement position is moved from the undipped solid region to a thick layer of liquid, the intensity of the Au 4f signal decreases sharply to background level as the Au electrode becomes covered by a thick liquid layer (extracted individual spectra in Figure 3c).

**Figure 4:**
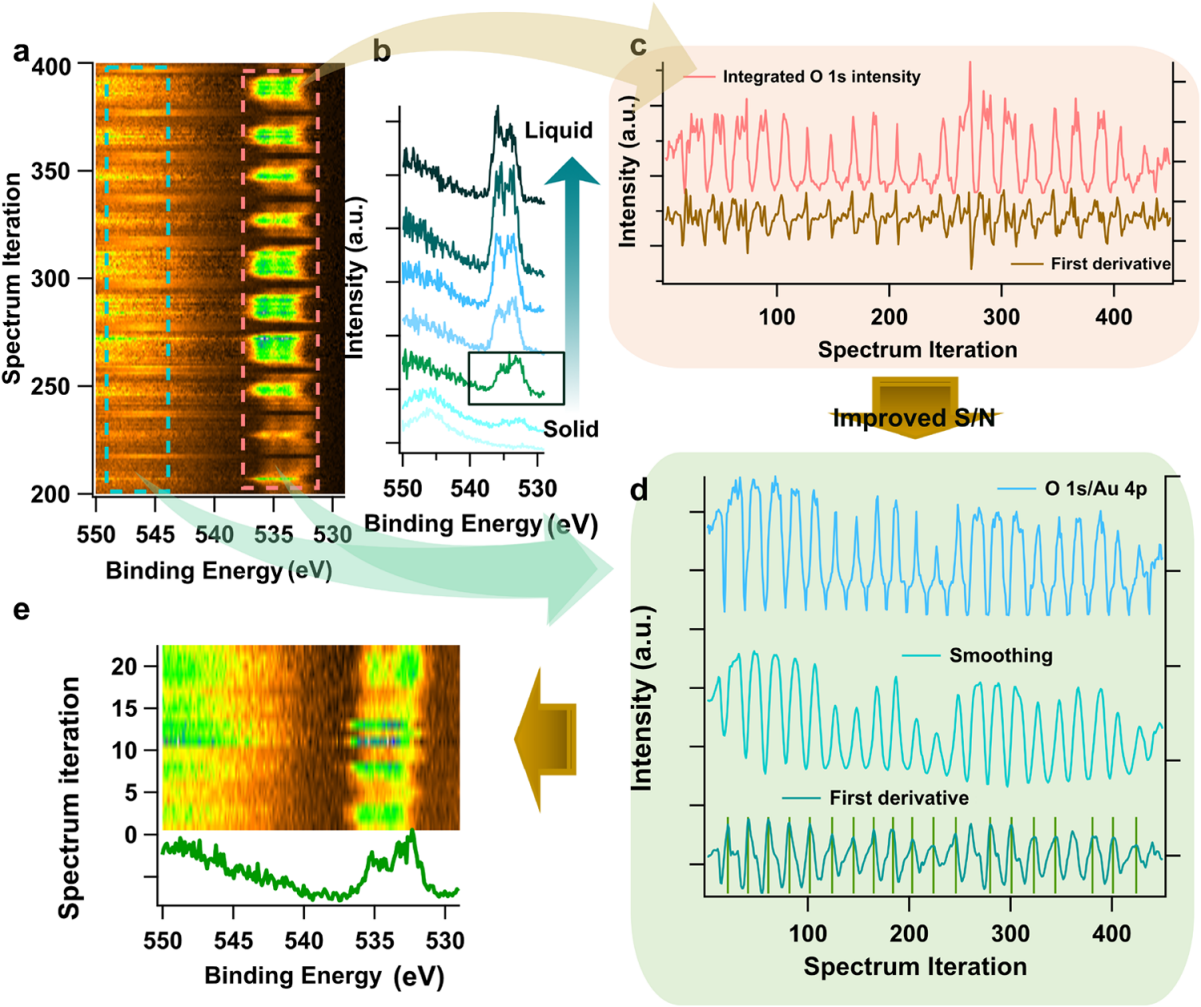
(a) The heatmap of O 1s spectra from the Au system after intensity calibration by aligning the background level, (b) the extracted spectra from one round of vertical scanning from solid to liquid (bottom to up), (c) the integrated intensity from electrolyte signal (536–532 eV) and its first derivative with respect to spectrum iterations, (d) improved S/N ratio of integrated intensity curve by normalizing the electrolyte O 1s intensity by the Au 4p intensity from the electrode (545–547 eV), followed by smoothing to reduce high frequency noise. The peak positions (green sticks) of its first derivative returns the iterations of interface featured spectra. (e) Display of the selected interface-featured spectra heatmap and one extracted spectrum.

While this transition is quick, the scanning still captured at least one spectrum which can be ascribed to the interface region in each round of vertical scans (middle in Figure 3c). The spectrum preserves the spectroscopic features of Au 4f from the electrode with attenuated intensity due to the liquid layer. This is recognized as the “interface-featured” spectrum and corresponds to the edge of the Au 4f image signal in the heat map (green line in Figure 3a). Multiple interface-featured spectra may exist in each vertical scanning cycle. However, the low spatial resolution of this set of measurements (180 μm between consecutive spectra) and relatively sharp liquid edge results in only one interface-featured spectrum per core level in most scanning cycles. Measuring only a single core level (described in the following sections) significantly improves the spatial resolution due to the exclusion of dead time during analyzer voltage ramping, but sacrifices chemical information from other core levels. A smaller beam size can substantially improve spatial resolution but at the cost of reduced intensity.

Each individual interface-featured spectrum alone displays a low S/N ratio making it difficult to do quantitative analysis or identify interphase compounds (e.g., in C 1s or O 1s core levels). Therefore, scanning between the liquid and solid regions for 25 cycles was performed in the Au system, which is expected to capture around 50 interface-featured spectra, enabling a reliable representation of interface features and strong statistics when summing up the interface spectra.

To automatically and accurately recognize and select all the interface-featured spectra among the mixed spectra in the heat map, distinct spectral features can be used as stamps. The choice of indicator depends on the liquid distribution behavior on the sample, the core level studied, the presence of spectral features or elements unique to a particular phase, and also on the nature of the background intensity variations while scanning at the solid or the liquid phase in fixed mode. The following section outlines two software-based spectra recognition methods and their applicability to different data sets. These methods also provide the opportunity to map the chemical distribution spatially and the liquid topography across the liquid edge, which is displayed in the form of a 3D map.

### 2.2 Software-Based Data Processing for Interface Spectra Recognition

#### 2.2.1 Interface-Spectra Selection Using Image Recognition

The trend in spectral intensity variation when scanning from dry solid to thick liquid regions arises from the attenuation of photoelectrons emitted from the solid with respect to the thickness of the liquid layer. Specifically, the photoelectron signal originating from the solid underneath decays exponentially with increasing liquid thickness, as described by

1 $$\frac{I_{s}}{I_{s,0}}=e^{-\left(d_{L}/\lambda_{L}\cos\theta \right)}$$

where *I*_s_ is the intensity of photoelectrons from the solid after penetrating the liquid layer to reach the analyzer, and *I*_s,0_ is the intensity of photoelectrons from the solid under ambient pressure conditions without the liquid overlayer. *d*_L_ represents the thickness of the liquid layer, λ_L_ is the IMFP of electrons in the liquid phases, and θ is the angle between the sample surface normal and the analyzer axis.^45^ Based on this relation, an interface recognition method is developed by identifying the pattern of intensity attenuation and the distinct intensity transition characteristic of the interface region when transitioning from the dry solid region to the thick liquid region. Depending on whether the core level exhibits features from only one phase (e.g., Au 4f only contains features from the solid and C 1s only contains features from the liquid in the Au system) or both phases (e.g., O 1s exhibits both electrolyte and transition metal (TM) oxide peaks in the LCO system), the identification method of the interface region can slightly vary. In the latter case, the intensity ratio of the solid- and liquid-specific peaks can be used to exaggerate the transition.

##### 2.2.1.1 Intensity Attenuation of a Single Feature

In the example of the Au 4f core level of the Au system, the Au peak intensity pattern is described by plotting the integrated intensity of the Au peaks (ranging from 83 to 89 eV) against the spectrum iteration in Figure 3d (pink curve). The curve reveals sharp intensity peaks emerging from the baseline periodically. The interface-featured spectra, as shown in Figure 3c, reveal a rapid drop in intensity from the previous spectrum, referring to a large change in the local liquid thickness based on eq 1. To locate these interface-featured spectra, a mathematical description is proposed, which correlates the interface positions to the inflection points of the integrated intensity curve where a rapid decrease/increase in intensity takes place.

To recognize the “interface-featured spectra” among the series of spectra, the first derivative of the integrated Au peak intensity is applied to quantify the rate of intensity change (curve in brown, Figure 3d), representing the instantaneous slope of the pink curve. The first derivative is often used in spectroscopy and imaging studies for identifying peak onset, etc.^52^ The positive and negative peaks in the first derivative correspond to the points of maximum rate of change in intensity. In this system, positive peaks reveal the highest rate of the Au intensity increase when scanning from thick liquid to dry regions, while the negative peaks exhibit when the scan returns from dry to thick liquid regions. The positions of the two peaks align with the sharp transition edges at lines A and B in the spectra heatmap (Figure 3b), whose spectral features meet the characteristic criteria of the interface (see positive-peak selections in Figure S4).

An algorithm was developed using the software Igor Pro to systematically extract all the interface-featured spectra from the heatmap by identifying the local maxima and/or minima in the first derivative. The algorithm employed a multipeak search approach by identifying the peak (defined when the second derivative is equal to zero) in a fixed data window before jumping a predefined distance from the identified peak position and repeating the search. The indices of the selected peaks correspond to specific spectrum iterations, which are then used to extract the relevant spectra from the heatmap.

Figure 3e displays the selected interface-featured spectra. An example of a selected spectrum (green) exhibits the same spectral features as the interface spectrum displayed in Figure 3c. The selected 25 spectra show variations in intensity to some degree, revealing the location of the selected interface spectra had slightly different liquid thicknesses on each scanning cycle. This may be attributed to the limited spatial resolution obtained when cyclically acquiring spectra from all three core levels, which reduces the chance of capturing the interface region in the Au 4f spectra. Alternatively, it may also indicate variations of the liquid edge shape in different scanning cycles, which will be discussed extensively in subsequent sections.

##### 2.2.1.2 Double Features Contrast

For the O 1s spectra recorded together with Au 4f and C 1s in the Au WE system, the dominant signal in the heatmap, unlike the Au 4f spectra, originates from the liquid phase, specifically the PC/LiClO_4_ electrolyte. This is observed as peaks in the binding energy (BE) range of 536–532 eV (Figure 4a). In addition, for the spectra from the dry solid region (bottom), a broad peak centered at 546 eV is exhibited which is assigned to Au 4p_3/2_. Thus, by extending the BE range for O 1s, spectral contributions from both the solid phase (Au 4p) and the liquid (O 1s) phase can be captured within a single spectrum. However, in this same energy range with Au 4p acquired from the liquid phase, the background level continues rising toward higher binding energies (Figure 4) (attributed to the O 1s satellite located approximately 15 eV above the main peak Figure S5, survey), complicating intensity driven data recognition from the Au 4p_3/2_ peak. Therefore, the method is applied to the O 1s peaks from the liquid phase.

By integrating the intensity of the electrolyte signal (536–532 eV) and plotting this against the spectrum iteration, the resulting intensity curve exhibits a low S/N ratio (Figure 4c). In some cycles, shoulders appear during intensity decrease/increase rather than the sharp transitions observed for Au 4f (Figure 3b). These features result in high frequency noise and reduced contrast in the first derivative, making peak searching using the algorithm challenging as there appear to be many local minima and maxima per cycle (Figure 4c). This feature also suggests the presence of an extended thin liquid layer with nearly constant thickness near the upper edge of the liquid (Figure S6). However, these shoulder features captured in the O 1s spectra are absent in the Au 4f spectra in Figure 3d even though the Au 4f signal from the solid is essentially a reciprocal expression of the O 1s signal from liquid. Note that the O 1s intensity originating from liquid as the top layer phase is more sensitive to subtle variations in local liquid distribution as there is no overlayer to scatter the O 1s electrons. The attenuation of their intensity only comes from the thinning and the disappearance of the liquid. In contrast, the photoelectrons from Au penetrate through a liquid layer, and even a thin layer can cause significant inelastic scattering, leading to highly attenuated intensity and little contrast to the thick liquid region.

Given the opportunity that both solid (Au 4p at 546 eV) and liquid phase (O 1s at around 535 eV) are present in the same spectrum, to enhance the contrast of the O 1s signal intensity between dry solid and thick liquid regions, a double-feature-recognition method is applied where the Au 4p_3/2_ signal is incorporated into the image recognition. When the Au 4p decays exponentially with the liquid layer thickness *d* increases (eq 1), the O 1s intensity at the same time increases exponential (until the *d* reaches probing depth limit), by dividing the electrolyte O 1s integrated peak intensity by that of Au 4p_3/2_ (Figure 4c, upper), an increased contrast of the O 1s intensity curve is obtained (Figure 4d upper). However, shoulders are still present in some cycles, resulting in multipeak features in the first derivative despite an improved S/N ratio (Figure S7). This indicates the need for further refinement in order to successfully identify each peak in the first derivative.

Smoothing is further employed which is frequently used for preprocessing in digital signal data or spectra to reduce the high frequency noise or unwanted features.^53^ The Savitzky-Golay (S-G) moving-average smoothing is applied using a polynomial fit of the data (see method).^53–55^ Smoothing of the integrated intensity of O 1s/Au 4p eliminates shoulder features and fluctuations in the high intensity region where the liquid layer might be thicker than the probing depth (Figure 4d, middle). The first derivative returns a single negative and positive peak in each cycle with fewer high-frequency spikes (Figure 4d). Note that here the positive peaks reveal the interface positions when scanning from the dry region to the thick-liquid region, as was the case for the negative peaks for Au 4f. The selection of spectra obtained from identifying the positive peaks (Figure 4e) exhibit the same spectral features as the interface-featured spectrum in Figure 4b (marked by a black box). The selection obtained from the negative peak indexes (Figure 5b yellow) was also seen to have identical spectral features, demonstrating the effectiveness and reliability of the interface-spectra selection.

**Figure 5:**
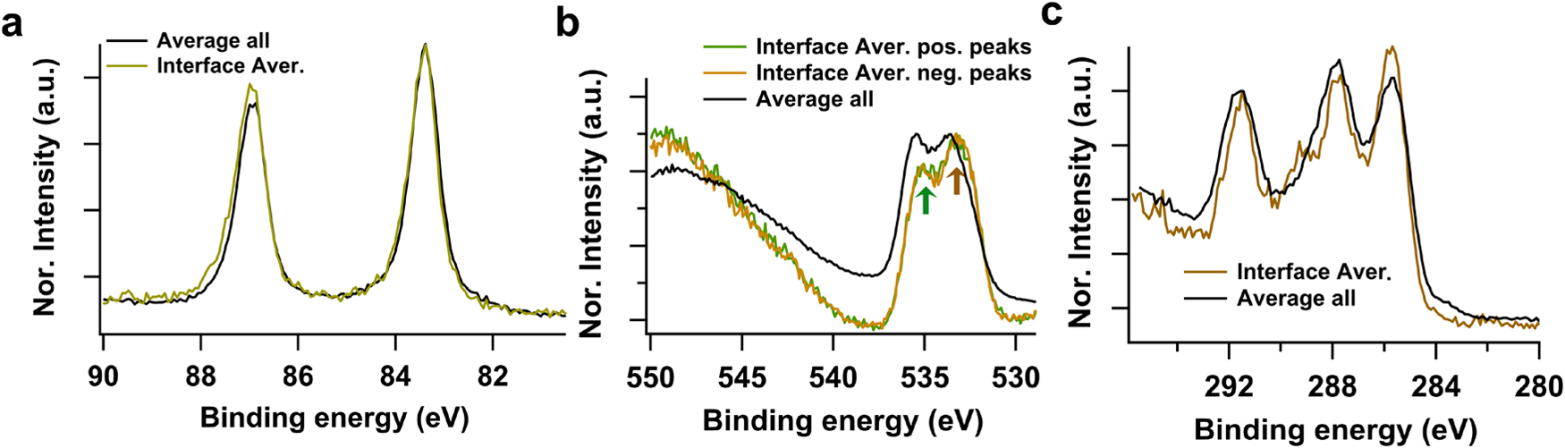
Comparison between the averaged, algorithmically selected interface-featured spectra (dark-yellow/green) and the averaged spectra of all spectra (black) of (a) Au 4f, (b) O 1s, and (c) C 1s. For spectral shape comparison, the intensities between the spectrum of average-all and interface selection are normalized to the highest peak intensity for Au 4f and O 1s, and to the carbonate peak (highest BE peak) for C 1s. An energy shift is also observed in C 1s between the average of all spectra and the interface spectrum. Note that a BE shift of −0.27 eV is applied here to align the peak positions of the two spectra for peak shape comparison.

##### 2.2.1.3 Comparison between Interface Selection and Nonselection

To analyze the effect of the interface selection with this method, a comparison of each core level between the spectrum acquired via the average of the selected interface-featured spectra and the spectrum acquired via a simple average of all spectra in the series without discrimination of spatial variation of the liquid layer is given in Figure 5.

In Au 4f (Figure 5a), the averaged interface spectrum (green) shows nearly the same line shape as the average (black) of all spectra, as well as the spectrum from only the dry solid region (Figure S8). Slightly more asymmetric peaks, broadened on the high energy side of the interface spectrum are observed, possibly due to the increased energy-loss of photoelectrons from the liquid overlayer. This asymmetry is less obvious for the average of all spectra as the signal primarily consists of contributions from the dry solid region. The intensity ratio of the Au 4f_7/2_ and 4f_5/2_ is around 0.82 and 0.86 for average of all spectra and interface-selected spectrum, respectively. The slightly larger deviated peak ratio from the spin orbital splitting (0.8) in the interface-selected spectrum is possibly due to its larger influence from the background shape as well as the energy loss electrons on the high BE side.

In the O 1s spectra (Figure 5b), the average of all spectra presents a high background level on the high energy side of the electrolyte peak, which is absent in both the dry-solid and liquid spectra (Figure 4b). In the high energy region around 546 eV, the average-all gives an artificial shape with lower relative intensity than Au 4p feature in the dry-solid spectrum but higher than the O 1s satellite in the liquid spectrum. A clear difference in the ratio of the two main O 1s peaks is observed between the average-all and interface-featured spectra (green and brown arrows). The main peaks consist of the C–O component from PC solvent at the high BE peak, and the C=O component at the low BE peak where it also contains the main part of the LiClO_4_ component^42^ (see a deconvoluted O 1s spectrum in Figure S9). A relatively higher intensity at the low energy side (brown arrow) from the interface-featured spectrum may indicate a higher salt concentration at the solid/liquid interface than in the bulk liquid.

In C 1s, a significant peak shift of around 0.27 eV is seen between the two spectra. In Figure 4c, the peak energies are aligned to allow a comparison of the peak shapes. A narrower peak width is observed in the selected interface-featured spectrum compared to the average of all spectra. As the spectra in the full series see variations in peak energy and peak shape in O 1s and C 1s, broadening of the peak occurs when summed up. This peak broadening is especially visible for a single-component peak, such as the peak at 291.5 eV corresponding to the carbonate component in PC. Additionally, the interface-featured spectrum deciphers the minor compounds at the interface, which are masked in the average of all spectra by mixed features from dry solid and thick liquid, e.g., the extra peak at 289 eV in the interface-featured spectrum. As the applied voltage of 0.8 V is in the potential range where the formation of SEI is supposed to occur, this interfacial component might be an insoluble reaction/side reaction product deposited on the electrode surface as part of an interphase. More systematic analysis is underway to demonstrate the chemical identity and reactivity of this interfacial compound, however, this observation demonstrates the value of isolating interface features and the effectiveness of this recognition method.

The intensity attenuation method was also effectively employed on the LCO|LiClO_4_/PC system. The O 1s spectra present features from both PC electrolyte and TM oxide (Figure S10), providing a direct identification of the interface featured from a single core level. However, compared to the Au electrode, better wetting and fast adsorption of the PC liquid occurs on the LCO thin film. As a result, significant electrolyte intensity is consistently observed in the spectra across the entire scanning region even above the dipping position, after the system has remained in the chamber for several hours. Therefore, the selection process relying on intensity contrast, is enhanced by the ratio between the TM oxide and electrolyte peaks after smoothing (Figure S11a,b). The interface selections (Figure S11c,d) reveal that the TM oxide intensities are very low and show minimal contrast to the updipped region where the sample did not undergo reaction in the beaker and/or is not in equilibrium state.

Overall, the method successfully identifies and selects interface spectral features and is applicable to different systems. Recognition is dependent on the liquid distribution on the WE, which influences the intensity contrast transient from dry to wet regions. Thus, the choice of signal contrast and the parameters in the processing are required to be adjusted depending on the system and/or core levels of interest. As a fast and feasible approach, it brings possibilities to assess interface features on-the-fly. The ability to resolve buried minor interfacial compounds highlights the importance of isolating and analyzing the true interface chemistry.

#### 2.2.2 Interface Spectra Selection Using Energy Alignment

Using the intensity attenuation method, we note that for both Au and LCO systems, the selected interface-featured spectra still show variation in the spectral shape and peak position to some degree (Figures S11c,d and S12a,b), suggesting that the selected spectra are not in identical electrochemical potentials and/or chemical environments. The drawback of using intensity attenuation to select the solid/liquid interface feature is that there is always a selection for each round of scanning, corresponding to a rapid change of liquid layer thickness. The shape and thickness of the liquid edge on the surface varies with respect to space and time, as discussed above. Thus, the selected interface spectra may not necessarily come from measurement positions with identical liquid thicknesses. One consequence of this is described as follows. The potential difference between the electrode and electrolyte is mainly carried by the electrostatic potential drop in the Electric Double Layer (EDL) formed by the redistribution of ions at the interface (when no severe space charging occurs on the electrode).^56,57^ In the Guoy-Chapman model of the EDL, the electric potential drops exponentially with distance from the electrode/electrolyte interface (Figure 6a). When the probed liquid layer thickness is within the range of the EDL length, the photoelectrons emitted from different layer thicknesses will have different energies causing apparent BE shifts in the probed core level spectra.^57^ A sum of the core level spectra probed from different liquid thicknesses thus causes peak broadening, shifts, and/or asymmetry.^56,57^

**Figure 6:**
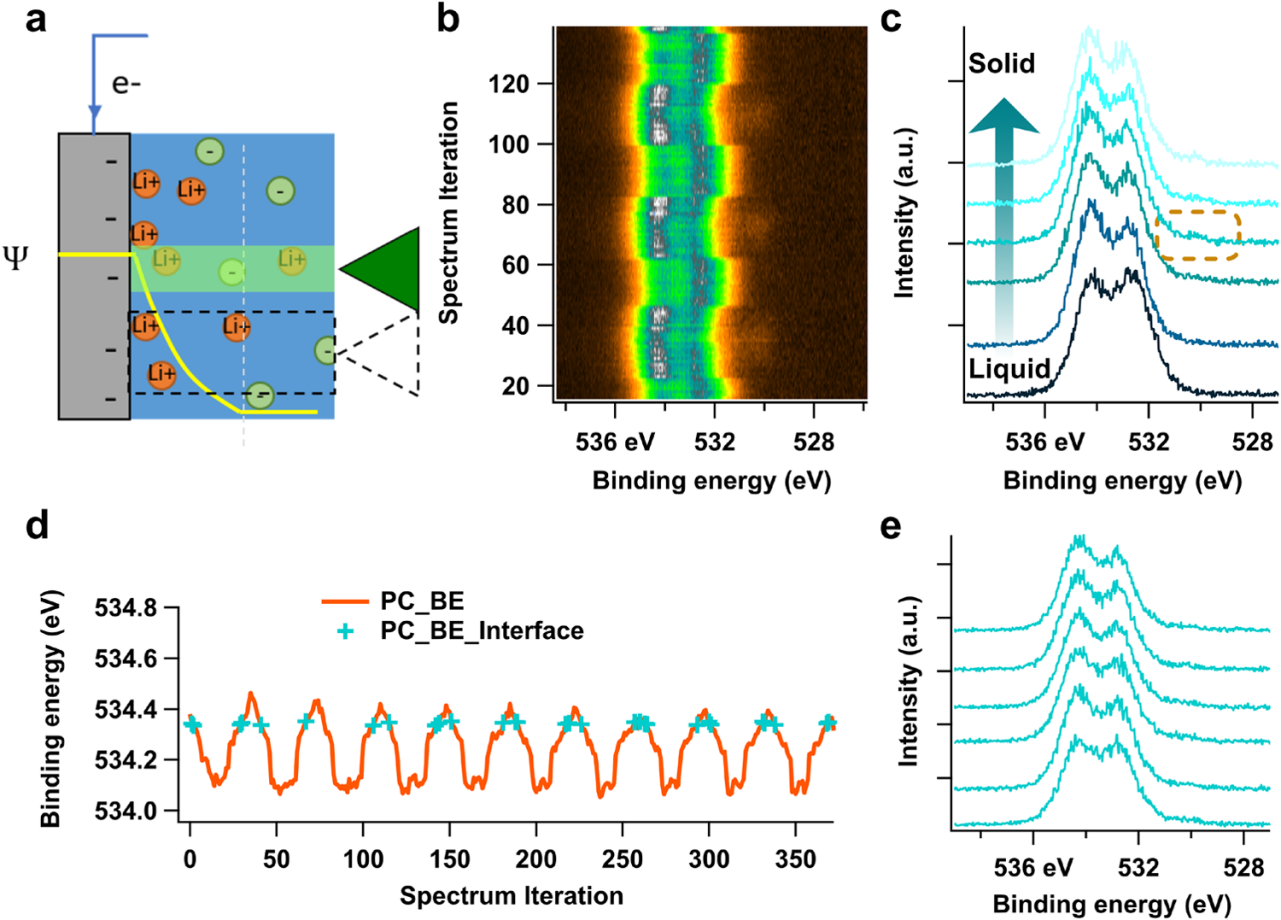
(a) Schematic illustration of the interface potential drop, causing the probed electrons to have different energies. Ψ refers to the electric potential of the phases. (b) O 1s spectra heatmap from the LCO system with (c) extracted spectra in a single round of scanning. (d) the fitted BE from PC electrolyte with blue marks from the selected interface spectra by BE recognition. (e) The selected interface-featured spectra showing the same spectral features and energies.

Figure 6b presents O 1s spectra from the LCO system under an applied voltage of 3.5 V vs Li^+^/Li. Obvious peak shifts from the electrolyte signal are seen in the heat map in each scanning cycle across the dry and thick-liquid regions. In line with the energy shifts, the spectra correspondingly exhibit changes in the relative ratios of the two main peaks from the electrolyte and the TM oxide (Figure 6c). To determine and analyze these peak shifts, curve fitting is applied to all O 1s spectra in the series using a pseudo-Voigt function combined with an autofitting algorithm developed in the Igor Pro environment with constraints in relative energy spacing, relative intensities and peak width (SI Note 2.2). The fitting parameters of the spectra, i.e., peak intensity, energies, and width, are collected in a matrix. Even though the low S/N ratio of the individual spectrum brings uncertainty for accurate peak intensities and width determination, the curve-fit gives satisfying results for peak positions. We found that the apparent BEs of electrolyte peaks in O 1s show periodic shifts to higher and lower energies between 534.46 and 534.07 eV when scanning between thick liquid and dry-solid regions. Because the measured BEs of the electrons from the electrolyte are connected to its electrochemical potential,^3^ this suggests that the electrolyte possesses varying potentials at differing vertical positions of the liquid layer (i.e., how far the measurement position is above the liquid reservoir). Based on the discussion above, the apparent shifts in BE with respect to the vertical position on the liquid edge are possibly linked to the electrostatic potential drop in the EDL which is in turn related to the liquid thickness increase from the liquid edge to the bulk. Another explanation is that there is an *iR* drop vertically in the meniscus (i.e., a potential difference between the electrolyte at the top of the liquid and the bottom, closest to the reservoir), which is expected to form during charge transfer reactions when a significant current is flowing through a thin layer of liquid.^43^ However, the latter explanation is unlikely in this setup as the measurement was conducted at steady state when the current had converged to a negligible value. Besides, the system under the applied voltage of 3.5 V is below the redox voltage plateau of around 3.9 V vs Li^+^/Li for LCO,^42,58^ therefore, no major delithiation/lithiation is expected to occur between the electrode and electrolyte. More studies are required to unravel the possible reasons for the peak shifts and determine the mechanisms. However, presence of the periodic BE shifts brings about the possibility for interface spectra recognition.

The close relation between the BE and local liquid thickness (and local chemistry) allows the regular variation in BE to be used for interface feature selection. We defined the interface-featured spectrum to be the first spectrum presenting a visible electrode feature (e.g., TM oxide peak) when scanning from the thick liquid up to the undipped LCO electrode. This ensures that the probed interface position has sufficient electrolyte thickness for electronic connection to the bulk, implying its electrochemical activity. By manually selecting one interface-featured spectrum from a single cycle of scanning (spectrum marked with a dashed box in Figure 6c), the PC peak position can be extracted and used as a stamp to select all the interface spectra from the full series with the same peak energy, where a narrow energy range 534.35- 534.33 eV is allowed. The selections correspond to the blue crosses in Figure 6d. The selected spectra (Figure 6e), exhibit minimal energy shifts, as expected. In addition, all spectra exhibit consistent spectral features regarding the TM oxide peak intensities and the peak ratios of the two main electrolyte peaks, suggesting the same chemical composition. It is demonstrated that the chemical nature and interface energetics are closely correlated and likely partially determined by the thickness of the liquid layer at the measurement location. By investigating the topography of the liquid edge and its variation in space and time, a better understanding of how the probing position and local liquid distribution can affect the spectroscopic outcome can be gained.

### 2.3 Spectro-Microscopic Imaging of the Liquid Edge

When both solid and liquid signals are simultaneously probed in a single core level spectrum, the thickness of the liquid layer can be estimated from the ratio of the liquid and solid peak intensities.^45,57,59^

2 $$\frac{I_{L}}{I_{s}}=\frac{n_{L}}{n_{s}}\times \frac{1-e^{-d/\lambda_{L}}}{e^{-d/\lambda_{s}}}$$

Where *n*_L_ and *n*_s_ are the atomic density per unit volume of the liquid and solid component, and λ_s_ is the IMFP of the solid. Here, in the LCO system, as the O 1s describes both the electrolyte and the electrode, the ratio of the fitted peak areas corresponding to the PC and TM oxide components can be used to infer the electrolyte thickness. To determine the liquid thickness *d*, Newton’s method is employed (see Experimental Section 5.4) as the equation is not analytically solvable.^60^ Using this approach, it is possible to obtain the liquid thickness at each measurement position in the O 1s spectra acquired via scanning APXPS. In Figure 7, the system was held at a voltage of 3.2 V vs Li/Li^+^. A total of 5300 O 1s spectra were acquired over a 2 mm × 3 mm region near the liquid edge (Figure S24). Translation from the spectral features to liquid thickness results in a periodic wave ranging from 7 to 12.5 nm (Figure 7a). By further correlating the recorded sample manipulator positions with the calculated liquid thickness for each spectrum, a spectro-microscopic image of the real-time liquid distribution across the sample surface is obtained (Figure 7c). This enables visualization of the topography of the liquid layer, thereby demonstrating the potential of APXPS to provide both the distribution of different chemical species and, simultaneously, topographical information at a nanometer-scale thickness resolution.

**Figure 7:**
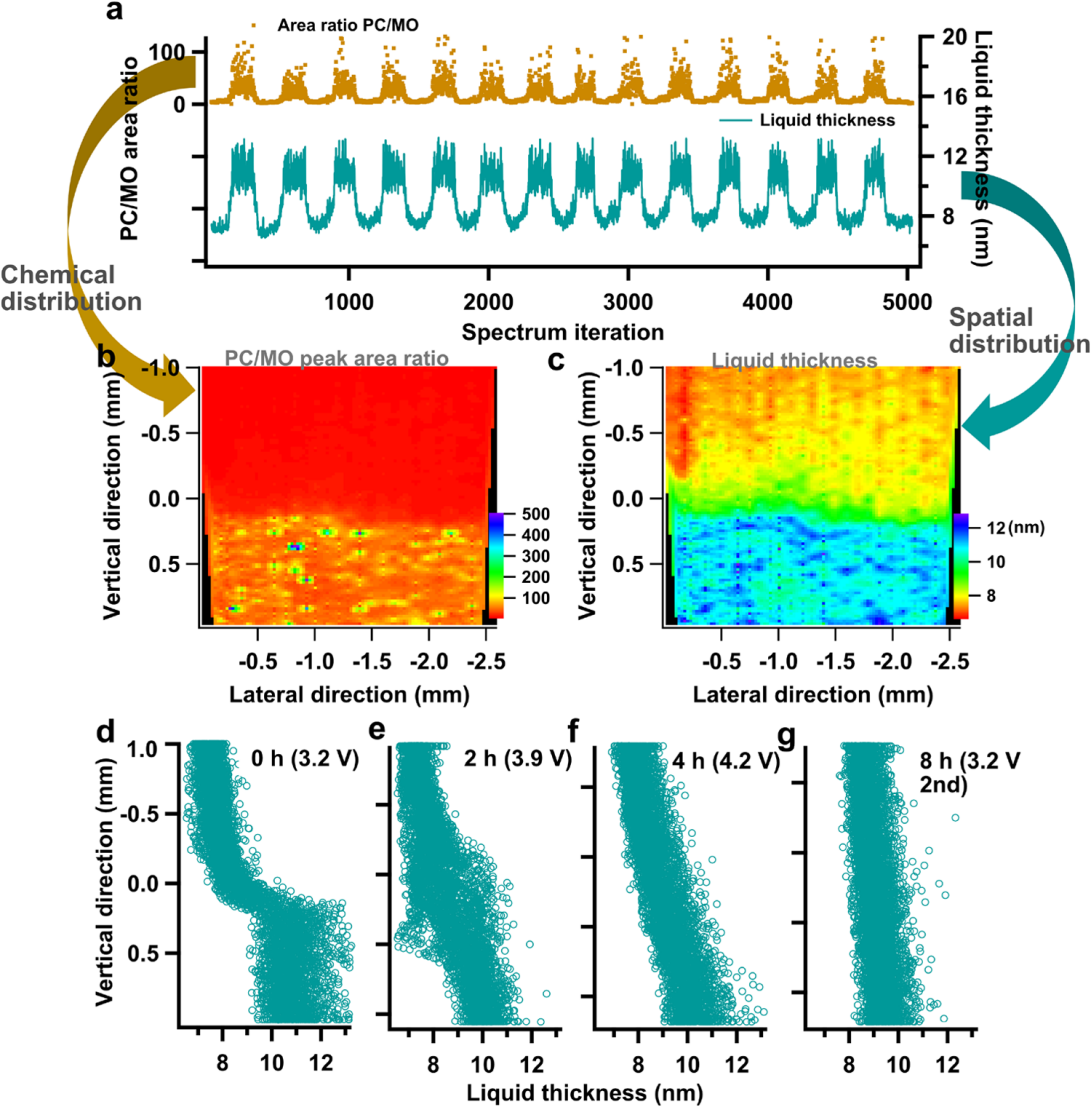
(a) The intensity ratio of PC to TM oxide obtained from curve fitting and the calculated liquid layer thickness for each spectrum, (b) The map of chemical contrast on the sample surface with spatial resolution. The signal is from the peak (area) ratio of PC to TM oxide. The *y*-axis refers to the relative height of the interface-feature position on the first scan cycle, same for panel (c–g). (c) The visualized liquid distribution on the sample from the liquid thickness vs the spatial coordinates on the sample, and (d–g) the side view of the liquid profile on the solid surface under different applied voltages, where the vertical coordinates of the measuring spot are plotted versus the liquid thickness. The time span between the first measurement (3.2 V) and the last measurement (3.2 V-second) is around 8 h.

The image of PC/TM oxide peak ratio (Figure 7b), which simply gives the chemical distribution of the surface, shows a sharp transition from the undipped region to the thick liquid region. The translated liquid thickness image exhibits a comparatively more gradual intensity distribution with distinct vertically separated regions, corresponding to an “undipped” region (red region, which still shows a liquid thickness around 7 nm possibly from wetting by capillary force or by surface absorption), an extended thin liquid layer (green region) followed by a thick liquid layer (blue region). Based on the exponential relation between peak intensity and liquid thickness (eq 2), an improved topographical resolution can be obtained by spatially plotting the calculated liquid thickness. An increased spatial sensitivity in the surface plane offers a more accurate representation of the actual liquid distribution compared to chemical contrast alone (Figure S13).

Side profiles of the liquid-edge shape obtained by plotting the vertical coordinates vs liquid thickness (Figure 7d) provide an alternative perspective of the liquid topography. Initially at 3.2 V, within the 2 mm probing length, an extended thin liquid layer approximately 7–9.5 nm thick is observed from the start. This is followed by an abrupt transition to saturation at an average of 11 nm with a wide range of variation between 10 and 13 nm. The profile resembles a step function rather than the commonly assumed meniscus shape.^61^ The probed minimum liquid thickness of around 7 nm suggests that the scanning does not reach a fully dry region, even though the uppermost measurement position is located 1 mm above the dipping level. This reflects the wetting behavior of the liquid on this thin LCO film sample. At the thick liquid position, the large variation of obtained liquid thicknesses may result from several factors beyond the actual shape of the liquid edge. First, the thickness of around 11 nm is close to the theoretical probing depth limit in liquid PC, estimated to be around 12 nm based on 3 times the IMFP in PC at a kinetic energy of 1.27 keV (Figure S14). Therefore, the intensity of the liquid signal will no longer increase when the liquid thickness is larger than the probing limit. Second, as the liquid thickness increases, the intensity from the solid becomes very low, possibly in little contrast to the background level. This potentially leads to inaccuracies in curve fitting, such as when the background noise is mistakenly recognized as a peak component. This also partially explains the large variation in the calculated liquid thicknesses in the thick liquid region. To mitigate this fitting uncertainty, a constraint was applied to the fitting parameter to ensure the TM oxide intensity remains ≥0. Any intensity below 0 is set to 0, and the corresponding data point is excluded. TM oxide intensities greater than zero return valid liquid thickness values below the probing depth limit as is defined by λ_L_ and λ_s_. Consequently, the shown thickness in the thick region may not reflect the true liquid shape, as the liquid layer exceeding the probing depth, where no solid phase signal is present, can not be accurately determined.

Furthermore, The shape of the electrolyte edge is important for the electrochemistry at the measured spot, and its dynamic nature is likely linked to multiple factors, e.g., applied voltages and time. The spectro-microscopic imaging enables the observation of the gradual wetting of the electrolyte on the electrode, as exemplified in Figure 7. A total time interval of 8 h was spent for this system to be conducted under a series of voltage steps, 3.2 V, 3.9 V, 4.2 V, and returning back to 3.2 V Li/Li^+^ (see electrochemical data in Figure S22). Each step was measured after redipping to replenish the liquid and to form a new dipping line. The 3D liquid distribution images (Figure S15) reveal the thick liquid region gradually diminishing, and the thin liquid region progressively expanding to cover the entire probed area. This gradual wetting is also clearly revealed in the side-view profile. Specifically, the average thickness of liquid in the thickest region decreases from 11 to 9.5 nm between the initial 3.2 V and final 3.2 V-second measurements. Meanwhile, the starting thickness at the uppermost probing position increases slightly from 7 to 8 nm and the overall gradient of liquid thickness-growth becomes less steep. While variation in liquid thickness may also be influenced by changes in the WE charge state under different applied potentials, the significant spread of liquid between the first and last measurement at 3.2 V, as well as the continuous spreading of liquid seen from both 3.2 V to 4.2 V and 4.2 V to 3.2 V-second suggests that this behavior is primarily attributed to time-dependent wetting of the porous electrode. This time-dependent effect on the liquid shape is also visible within the time scale of one voltage step, where the vertical location of the thick liquid region is seen to move downward, extending the thin liquid region 45 min after pulling up (Figure S16). The dynamic liquid distribution exemplifies the importance of being able to monitor the liquid geometry instantly for a correct interface feature selection and interpretation.

#### 2.3.1 Au/PC System

To compare the liquid edge distribution profile between different WE surfaces, the scanning APXPS imaging was applied to the Au system using the Au 4f core level. Since the core level only exhibits the solid phase signal, the liquid thickness can only be estimated using eq 1. Here *I*_s,0_ is assumed to be the integrated peak intensity obtained at the topmost scanning position, where the Au intensity remains constant, indicating a dry region. This assumption is supported by the O 1s spectra from the cyclic measurements (Figure 4a), which shows no PC signal at the highest measurement position. Therefore, the intensity ratio between the selected reference point *I*_s,0_ and that from the wetted region provides an estimation of the liquid thickness (Figure S17).

The obtained liquid distribution image reveals a much more abrupt wet/dry boundary with sharper transitions from the dry region to the thick liquid region compared to that seen with LCO (Figure 8a). Over the time span of 2 h between the two measurement series conducted at 2 and 0.8 V, no obvious temporal wetting above the dipping line was observed. This sharp wet/dry transition does not appear to be affected by the pulling velocity of the WE from the beaker either (Figure S18). Between the dry and thick liquid regions, a narrow band of thin liquid appears, approximately 150 μm wide. The side view profile shows a sharper step jump from the undipped region (Figure 8b) of nearly constant thickness to the thick liquid region with a saturated liquid thickness of around 9 nm, albeit with great variation. This thickness does not reach the theoretical probing depth limit. However, the shown value is not necessarily the actual thickness in the thick liquid region where the spectral feature from Au is not visible. Even though the error caused by curve fitting uncertainties as in the LCO system can be dismissed in this system, the lower saturation limit of liquid thickness is possibly caused by the background intensity at the thick liquid region, which is included as Au signal intensity in the calculation, returning a limited value of liquid thickness. The data points of uncertain liquid thicknesses where no Au signal presents in the spectra are marked gray (Figure 8b). The selection of the data points utilizes statistical analysis methods which identify the ranges of the regions where the Au spectral intensities attenuate to background levels (See Figure S19 and note).

**Figure 8:**
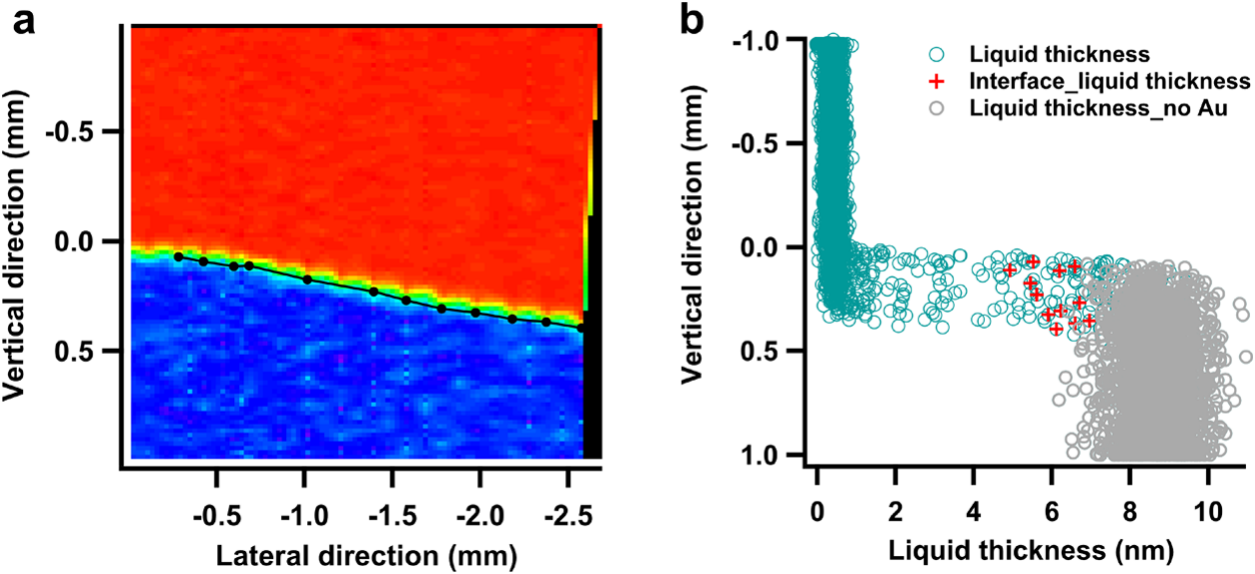
(a) Visualization of the liquid distribution on the Au sample and the locations of interface-featured spectra selected using the intensity attenuation method (dark line). (b) The side view of liquid thickness on the solid surface, where the red marks are the liquid thickness corresponding to the selected interface spectra. The gray circles correspond to the spectra data with no Au 4f feature observed when the local liquid is too thick.

In addition, a notable feature worth discussing is the narrow thin-liquid band between the dry and thick liquid. In this set of measurements, the vertical spacing between two adjacent spectra is 8–10 μm. However, with a larger beam size of 50 × 50 μm^2^, every five consecutive spectra contain overlapping information from the same beam spot. Thus, the effective spatial resolution is highly reduced. This raises the concern of whether the shown thin liquid band is artificially caused by the spatial resolution of the beam spot, specifically, whether the beam spot partially irradiates both the dry and thick liquid regions, resulting in a reduced peak intensity “acting” as a thin-liquid interface. To evaluate this, first of all, it is noted that the resolved thin liquid region from the Au 4f image is about 150 μm, which contains at least 3 beam spots with no overlap, confirming that the presence of thin liquid is certain. However, the effect of partially irradiating both dry and wet regions on the spectral features and how this influences the true dimensions of the resolved thin liquid region needs to be investigated.

To understand this better, two spectra were selected from a dry region and a thick liquid region respectively (Figure S20a), and linear combinations of them were used to simulate the partial beam overlap. The liquid phase does not contribute to the Au 4f spectral signal. However, due to the lower scattering cross-section in liquid phases, the background levels are typically orders of magnitude lower than in the solid phase, resulting in near-zero intensities. When the two spectra were combined with equal weights (0.5 each), the resulting spectrum retained the same shape as the original solid spectrum, with only a shift of intensity scale to half (Figure S20b). Even when the solid spectrum was weighted at 0.1 (and the liquid at 0.9, Figure S20c), the combined spectrum still preserved the original peak shape with a slightly elevated noise level in the background region. When further reducing the weight of the solid spectrum to 0.05 to have a comparable total intensity with the actual interface-featured spectrum obtained using the intensity-attenuation method, the spectral feature still showed obvious differences (Figure S20d). The selected interface spectrum exhibits a high noise level not only in the background but also at the peak itself. This differs from the simply combined spectrum, which maintains the smooth, solid peak shape with only background noise. This analysis also indicates that the actual width of the thin liquid region may be larger than the resolved 150 μm. Besides, as shown in Figure 5, the interface-featured spectra in both O 1s and C 1s show different chemical compositions from those of the bulk liquid and dry solid. These comparisons suggest that the interface-featured spectrum is not a simple linear combination of spectra from dry and thick liquid regions but comes from a true physical interface region of intermediate liquid thickness and potentially distinct chemical composition.

By appending the coordinates of the selected interface-featured spectra onto the liquid distribution image (Figure 8a dark line, and Figure 8b red markers), it is evident that these spectra are located at the transition between the thin and thick liquid region rather than the dry and thin liquid boundary. The corresponding liquid thickness is around 6 nm (Figure 8b). This further dismisses the possibility that the measurement represents a partial irradiation of both dry and thick liquid regions. Although the spatial resolution is limited by the beam size, which brings uncertainty in precisely defining the liquid shape, statistics over multiple scanning cycles assist in improving the accuracy.

## 3 Discussion

The relationship between the attenuation of the solid phase signal intensity and the thickness of the overlying liquid provides a distinct signature for identifying the solid/liquid interface. The developed interface recognition method using solid signal intensity attenuation exploits this relationship by identifying a sharp transition in the intensity of the spectral signal from the solid or liquid phase, which corresponds to a rapid change of liquid thickness. This method is efficient for interface identification and selection. It is a fast approach with little computational power required, which is beneficial for on-the-fly analysis. The method is effective independent of the electrochemical state of the sample, i.e., it can be used when the system is undergoing a varying applied voltage or current. However, for porous electrodes, gradual wetting over time can extend the thin liquid layer into regions on which no reaction have taken place or where the reaction is not equilibrated. As a result, the selected interface spectra features may vary between scanning cycles, showing different chemical compositions and energetics. Additionally, temporal and spatial variation in the thickness of the liquid at the edge can lead to peak shifts in the selected liquid phase spectra due to changes in electric potential with depth or height.

This variation led to the development of a BE recognition method which is based on the relationship between the apparent BE and the local electrochemical potential. Interface spectra selected using this approach exhibit consistent energetics and chemical environments as it is a selection of binding energy. Compared to the intensity attenuation method, BE recognition offers a more robust and accurate selection. The selection is independent of variations in sample material or liquid morphology, which is a better approach in a system with higher wettability between solid and liquid and larger sample inhomogeneity. It can possibly select more than one spectra from one round of scanning for statistics, which is beneficial for samples with limited spatial length and/or measurement time. Further, it is convenient for further data analysis, as by fitting the spectra, it directly profiles the chemical distribution of the scanned region. This enables the investigation of both chemical and potential evolution spatially, as well as liquid topography by further data treatment. On the other hand, the method has several limitations: (1) The low S/N ratio of the individual fixed-mode spectra in the XPS measurements can introduce uncertainty on peak position determination via curve fitting. (2) When the probed liquid layer thickness falls within the EDL length, the resulting peak is a convolution of components with different energies, and peak broadening may vary with probing position during scanning. This complicates automated curve fitting, where excessive degrees of freedom in the fitting algorithm can lead to a failure in finding a local minimum with correct chemical and physical meaning. (3) As this method captures the BE shifts, it thus can not be applied to the core levels from a grounded conductive WE (e.g., Au 4f in this study) as no BE shifts are expected regardless of the applied voltages. (4) Curve fitting as a preprocessing method is computationally heavy and time-consuming.

Scanning APXPS enables spectro-microscopic imaging which directly visualizes liquid distribution near the liquid edge with both chemical and topographical information. In the LCO system, a liquid layer thickness ranging from 7 to 11 nm is observed across a 2 mm vertical scanning range in the near-liquid edge region. The thickness profile vs vertical position is characterized by a thin liquid layer with a nearly constant thickness of around 7 nm, followed by a sudden step like transition to a thick region of approximately 11 nm. Gradual wetting of the electrolyte on the LCO surface is significant, where the liquid distribution evolves from a sharp transition between thin and thick liquid to a linear increase of thickness vs vertical position. In contrast, the Au WE surface exhibits an abrupt liquid thickness transition with minimal temporal wetting. Between the clear separation of the dry and wet regions, a narrow band of thin liquid, 150 μm wide, is observed. As the width is about three times the beam size, we dismiss the possibility that the presence of both solid and liquid signals is an artifact of the spatial resolution. Furthermore, the selected interface spectra are consistently located at the transition between the thin and thick liquid regions, which also alleviates the concern of a mixed dry/thick-liquid overlap. Additionally, due to the large difference in intensities between liquid and solid phases, a spectrum resulting from partial photoemission from both dry and thick liquid regions would predominantly reflect the solid phase feature. Therefore, the presence of distinct interface spectra supports the conclusion that the beam probes a true interface region. The sharp liquid edge observed in the Au sample compared to LCO highlights the influence of the surface morphology of the WE and wettability between the solid and liquid materials on the liquid distribution in the dip-and-pull setup. On one hand, a porous surface like LCO provides a larger region of thin liquid to access the solid/liquid interface, but it also promotes rapid transport of liquid up the electrode beyond the dipping line through pores or cracks, burying the solid phase quickly, which introduces problems for XPS measurements. On the other hand, a sharp solid/liquid boundary on the Au surface suggests poor wettability of PC on Au and a narrow range over which the interface can be accessed through a thin liquid layer. This underscores the importance of considering the solid/liquid affinity when selecting materials for a dip-and-pull measurement.

The methods presented in this study are broadly applicable to any solid/liquid interface investigation and are particularly valuable for operando studies of battery systems. Visualizing actual charge transfer mechanisms and real-time formation of decomposition products can only be done via direct probing of the solid/liquid interface. The difference in liquid distribution at the liquid edge between a flat metallic Au surface and thin-film LCO reveals the influence of surface morphology and solid/liquid wetability on the successful application of the dip-and-pull method. It also indicates the barrier to applying this setup on a true composite battery material with the current setup. As the dip-and-pull setup differs geometrically from a sealed battery cell, concerns surrounding the relevance of this model system to real battery systems arise. However, by carefully examining how the geometry of the liquid distribution affects the chemical and energetic environment at the interface, we can extract new, fundamental insights into reaction mechanisms and identify key factors influencing reactions and side reactions, while minimizing the influence of differences in experimental configurations.

## 4 Conclusion

A new methodology for investigating an active electrode/electrolyte interface in the operando XPS dip-and-pull geometry is presented. The method combines scanning APXPS along a programmed path across the liquid edge, with software-based spectroscopic interface recognition algorithms. The algorithms are demonstrated with two methods which recognize interface-featured spectra based on either the fundamental of intensity attenuation or liquid feature BE shift near the top edge of the liquid layer. The combination of the two methods provides a feasible and robust approach for on-the-fly interface feature analysis as well as a thorough spectroscopic mapping of the probed region. This approach further enables microscopic imaging with both chemical and topographical information of the liquid edge, revealing the influence of liquid edge geometry on interface features, improving sample control and data interpretation. The methods presented in this paper are applicable to a large range of solid/liquid interfaces, especially for battery systems, which advances the ability to understand true interfacial chemistry/energetics in real-time, and resolve minor interfacial compounds and their evolution during electrochemical reactions.

## 5 Experimental Details and Analysis Methods

### 5.1 Materials and Chemicals

The investigated LCO WEs were sputter deposited thin films with 300 nm thickness (5 sccm Ar, 20 mTorr, 80 W, base pressure 7.7 × 10^–7^ mbar). The substrate was alumina with a sputter deposited Pt layer on-top. Au WEs were prepared by sputtering Au for 4 min at 70 mA on copper plates (11 mm × 2 mm × 50 mm), which corresponded to a roughly 200 nm thick Au layer measured with atomic force microscopy. The electrolyte used for both systems was 1.0 M LiClO_4_ (Sigma-Aldrich) in propylene carbonate (Sigma-Aldrich). Composite film lithium titanate (LTO, Posco GS Materials) slurry coated with CMC (sodium carboxylate cellulose) and carbon black (Imerys & graphite carbon, C.NERGY SUPER C65) in 9:0.5:0.5 mass ratio was used as a counter electrode (CE) and reference electrode (RE) for the LCO system. Composite film lithium iron phosphate (LFP) coated with CMC and CB was used as CE and RE for the Au system, both precycled in half cells to their plateau potentials at 1.55 V vs Li+/Li for LTO, and 3.43 V for LFP.

### 5.2 Operando APXPS Electrochemical Setup

The experiments in this paper were conducted at HIPPIE beamline at MAXIV. A photon energy of 1800 eV was used for all systems in this paper. The SPECS hemispherical analyzer was configured with pass energies of 100, 150, 170, 180 eV for different core levels and a slit of 0.8 mm giving a theoretical analyzer resolution of 0.2–0.36 eV. The beamline energy resolution at 1800 eV photoenergy and 5 μm exit slit is around 0.2 eV.

The Operando APXPS experiments conducted in this paper use the same three-electrode dip-and-pull electrochemical setup as in our previous works.^42^ Voltages are applied between WE and RE and current flows between WE and CE. A closed circuit was always maintained throughout the dip-and-pull APXPS measurement. A solid/liquid interface can be accessed when the electrodes were pulled only partially from the beaker to expose a thin liquid film to the analyzer. Near the top edge of the liquid film, the solid and liquid can be probed simultaneously, which was then declared as an interface position. The WE was grounded to the analyzer. A BioLogic potentiostat SP200 was used in floating mode for simultaneous electrochemical control during the APXPS measurement. A potential control experiment was conducted with voltage applied between WE and RE. The electrical contact of the probed liquid was confirmed by the shift of liquid core level spectra line position when ramping the applied voltages. All the voltages values presented in the paper are converted to vs Li^+^/Li despite the REs in the systems being LTO and LFP.

The chamber pressure was maintained at the vapor pressure of PC at 0.2 mbar throughout the experiment.

The motion of the three electrodes and the beaker was controlled by a 4-axis manipulator. During scanning APXPS, the beaker and electrodes were moved simultaneously to keep a fixed meniscus length.

### 5.3 Smoothing

Savitzky-Golay Smoothing and Differentiation is used in the intensity attenuation recognition method. The smoothing calculates a polynomial fit of a chosen order, *x*. For a given signal measured at N points and a filter of width, *w*(*w* ≥ (*x* + 1)), this model smooths the curve by polynomial fitting of a selected box of data as the filter is moved across the data. The width of the data box is often an odd number to ease the calculation. The calculation returns the center point of the polynomial fit in the selected box. The data box moves along the data set after completing one calculation.

### 5.4 Layer Thickness Linear Approximation

Newton’s method is a root-finding algorithm. By moving the intensity ratio term to the right side of the equation, a function *f*(*d*) is given as

3 $$f\left(d\right)=-\frac{I_{L}}{I_{s}}+\frac{n_{L}}{n_{s}}\times \frac{1-e^{-d/\lambda_{L}}}{e^{-d/\lambda_{s}}}$$

Using an initial guess of the root, *d*_guess_

4 $$d_{1}=d_{\mathrm{guess}}-\frac{f\left(d_{\mathrm{guess}}\right)}{f^{\prime }\left(d_{\mathrm{guess}}\right)}$$

returns a root of the linear approximation of *f*(*d*) at *d*_guess_. Continuing the calculation of the same root starting from *d*_1_ and the resulting roots *d*_*n*_ from the linear approximations at *d*_*n*_ until it reaches the required convergency limit or iteration numbers, the algorithm completes its search for the root of the function (Figure S21).

### 5.5 Interface Recognition Algorithm

The code for the interface recognition approaches was written in Igor Pro, and was uploaded in github: https://github.com/qianhuiliu-git/apxps-Interface-recognition.

## Supplementary Material



## Notes

Additional spectra analysis methods, numerical analysis, SEM images, extra APXPS measurements data, and electrochemical data (PDF)
