# Supplementary material for: A Simple Approach for Operando Interface Probing for Batteries: Combining Scanning APXPS with Spectroscopic Recognition
Source: ACS Appl Mater Interfaces. 2026 Mar 18;18(12):18460–74. doi: 10.1021/acsami.5c25272 (PMC13051467; doi:10.1021/acsami.5c25272)
Supplement: Supplementary file 1 [file am5c25272_si_001.pdf]

Supplementary Information

A simple approach for operando interface probing for batteries: combining scanning APXPS with spectroscopic recognition

Qianhui Liu[1,+], Laura King[1,+], Helena Wagner[2], Alenka Križan[3], Laurin Derr[4], Katie L. Browning[5], Gabriel M. Veith[5], Tove Ericson[1], Robert Temperton[6], and Maria Hahlin[1,2,*]

[1]Department of Chemistry-Ångström Laboratory, Uppsala University, SE-75120 Uppsala, Sweden
[2]Department of Physics and Astronomy, Uppsala University, SE-75120 Uppsala, Sweden
[3]National Institute of Chemistry, Hajdrihova 19, 1000 Slovenia
[4]Institute of Physical Chemistry, Karlsruhe Institute of Technology, 76131 Karlsruhe, Germany
[5]Chemical Sciences Division, Oak Ridge National Laboratory, Oak Ridge, 37831 Tennessee, USA
[6]MAX IV Laboratory, Lund University, 225 94 Lund, Sweden
[*]Corresponding authors: maria.hahlin@kemi.uu.se
[+]These authors contributed equally to this work.

1 Figures

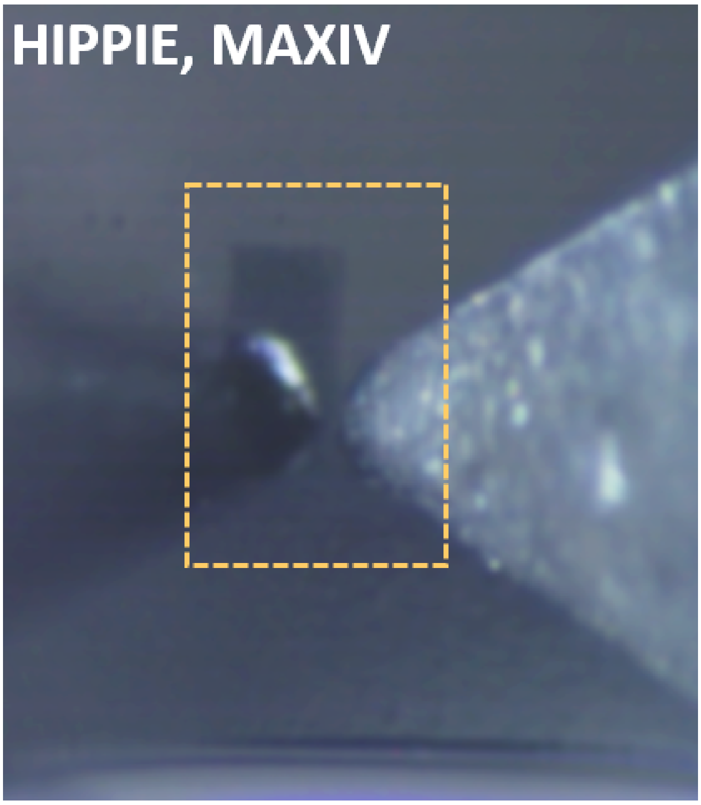

Figure S1: Liquid strips formed in front of the nozzle when the sample manipulator moves vertically during the measurement.

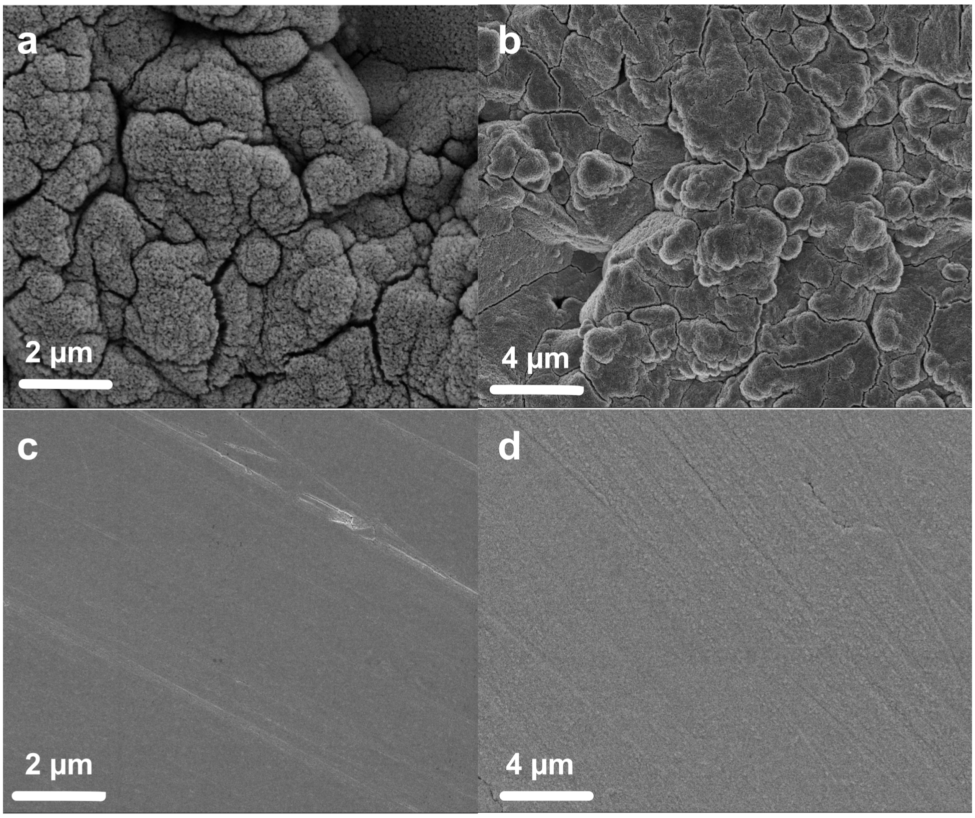

Figure S2: Scanning electron microscopy (SEM) of a), b) LCO thin film with 300 nm thickness, and c), d) Au thin film surface. The acceleration voltage is 5 kV. The a) and c) uses 10 k magnitude with It's observed with cracks and pores of around 50 nm width while the Au surface is more smooth with much smaller grain size.

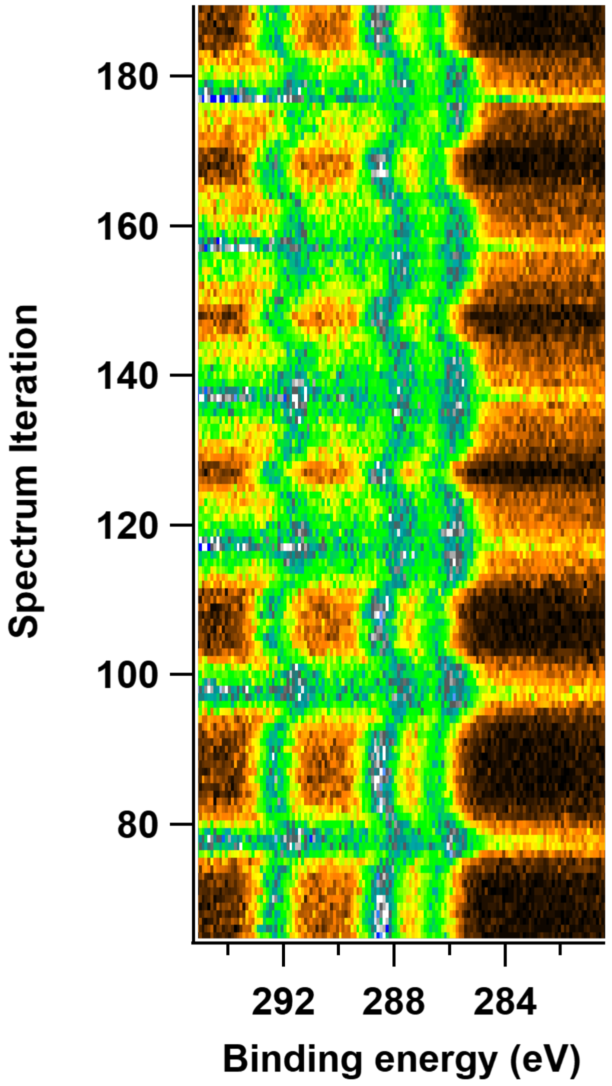

Figure S3: The heatmap of 130 out of 500 C 1s spectra measured at the interface with scanning APXPS across the dry Au WE and thick $LiClO_4$/PC electrolyte.

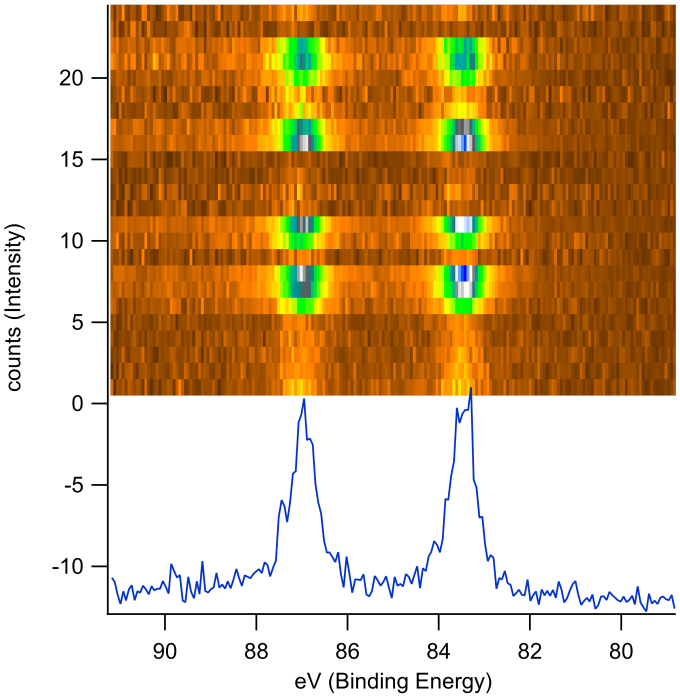

Figure S4: The interface-featured spectra of Au 4f from the selection of positive peaks in the first derivative wave. The spectral features represent same interface features with the negative peak selections.

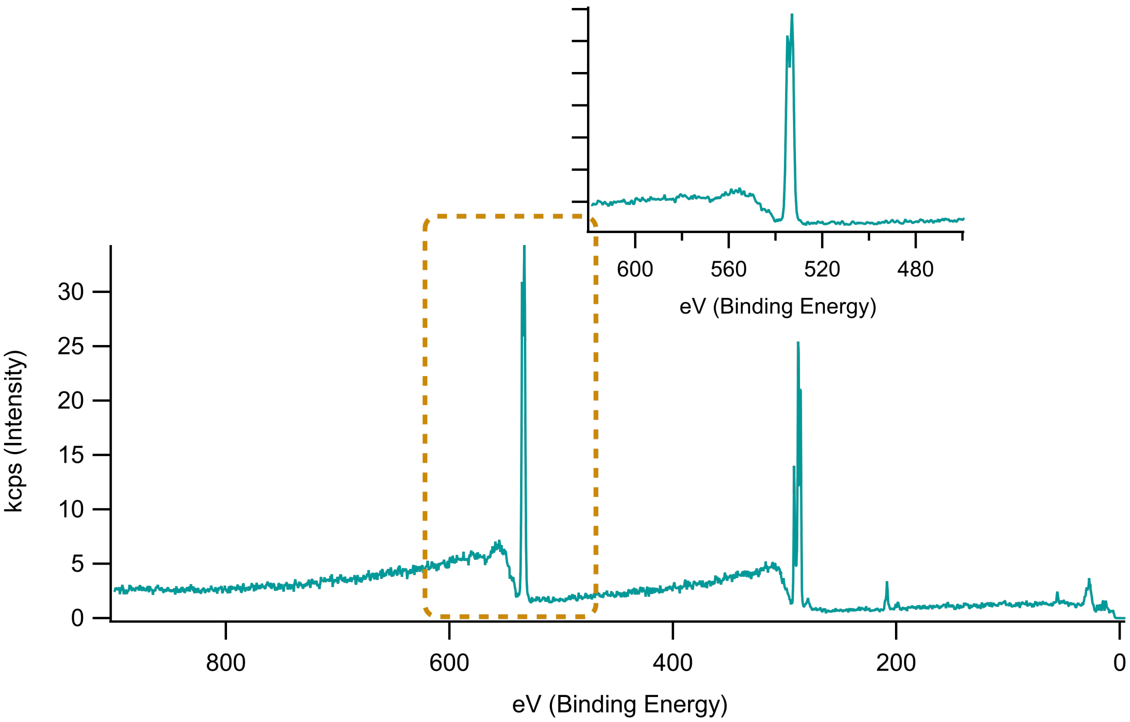

Figure S5: Survey spectrum for $LiClO_4$/PC liquid phase. The O 1s has a satellite feature at 559 eV (inset) which rises the intensity from 530-560 eV to give a high level of background.

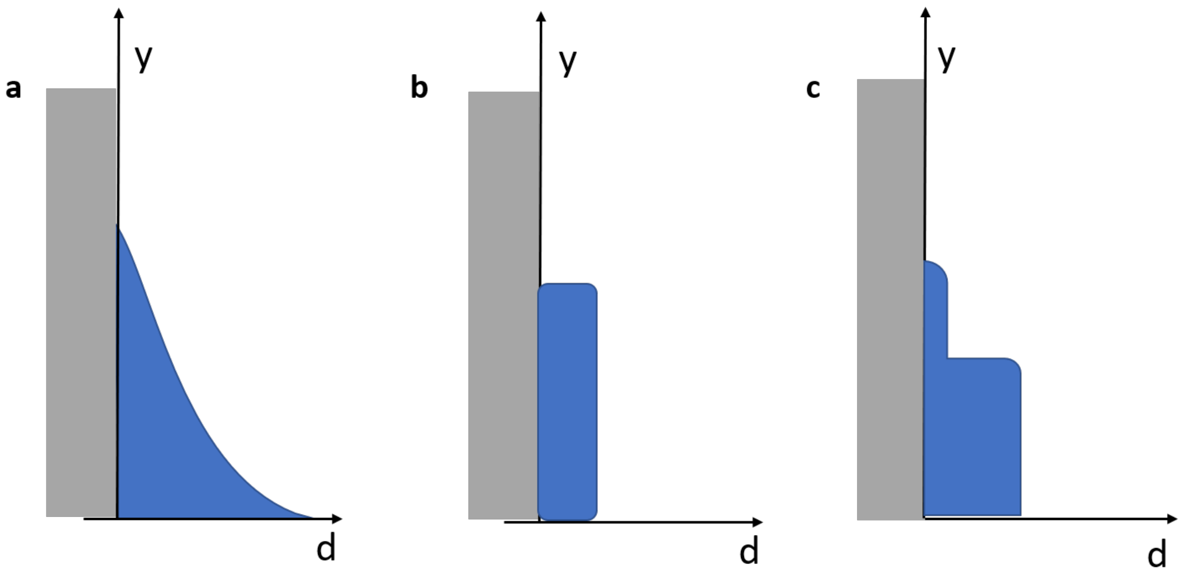

Figure S6: Schematic illustration of liquid layer shapes on the sample. a) The commonly assumed concave meniscus shape produced by surface tension. b) When the affinity between the liquid and solid is low, i.e. the contact angle between the liquid and solid is larger, the interface region is a sharp transition between dry solid and thick liquid. c) A liquid edge with an extended thin layer to form an "interface region".

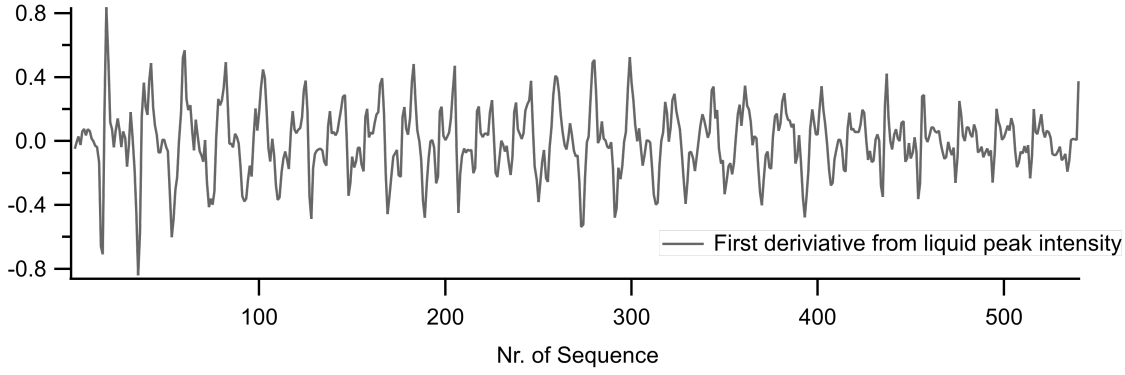

Figure S7: The first derivative from intensity of electrolyte PC peak in contrast to Au 4p intensity. The high frequency noise is reduced but multi-peak feature still presents.

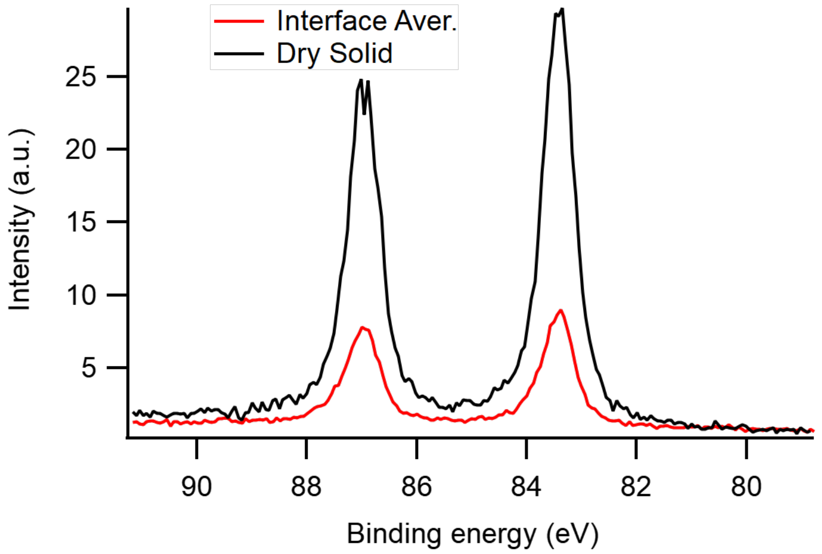

Figure S8: The comparison of Au 4f spectrum between one spectrum from dry-solid region and the averaged interface-featured spectrum.

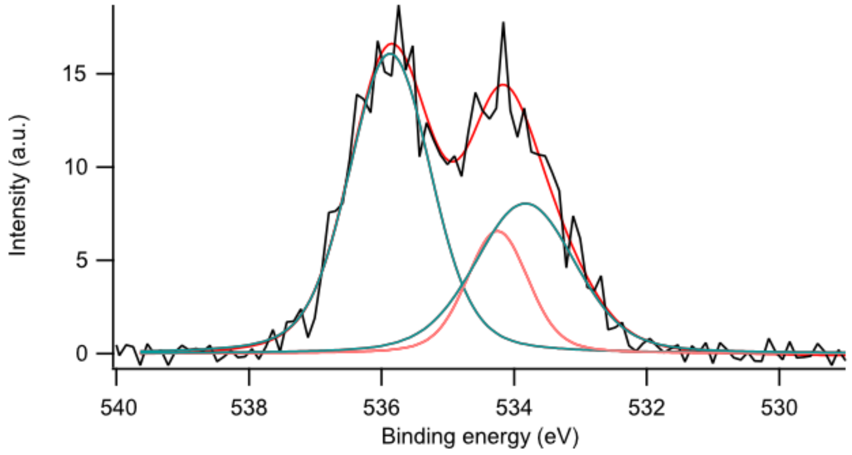

Figure S9: Deconvolution of O 1s from Au |$LiClO_4$/PC system. The peak ratio between the C-O and C=O components (blue) from PC solvent is fixed to be 2:1 with an energy spacing of 1.8 eV. The peak in pink is attributed to $LiClO_4$, with a fixed energy spacing of 0.42 eV between the C=O component.

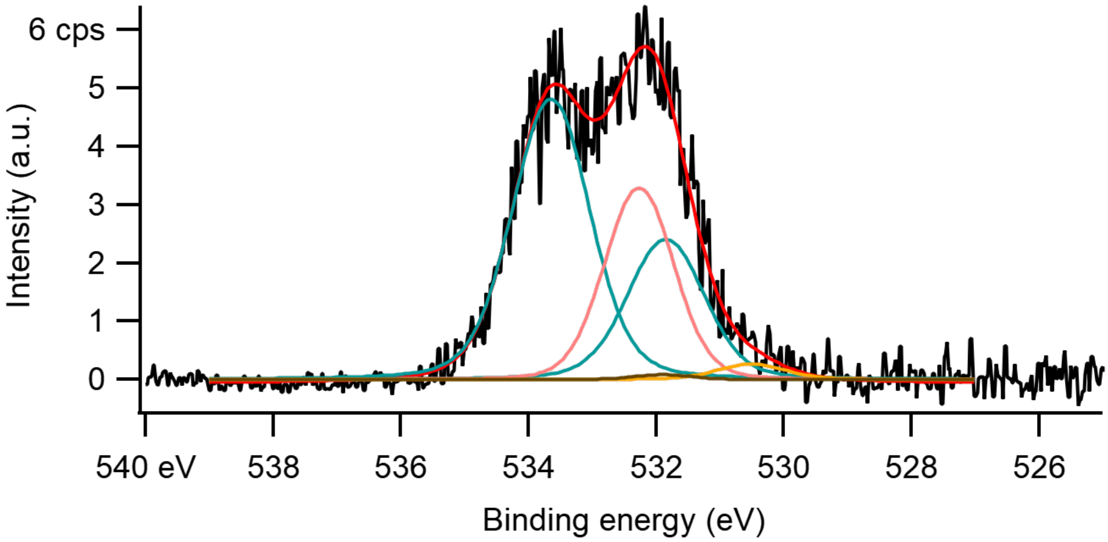

Figure S10: The O 1s from LCO|$LiClO_4$/PC system, and its curve fitting. The low S/N, and the complicated surface oxide results in uncertainty of peak intensity fitting for TM oxide peak, thus reduces the accuracy of the liquid layer thickness deduced from the liquid/solid intensity ratio.

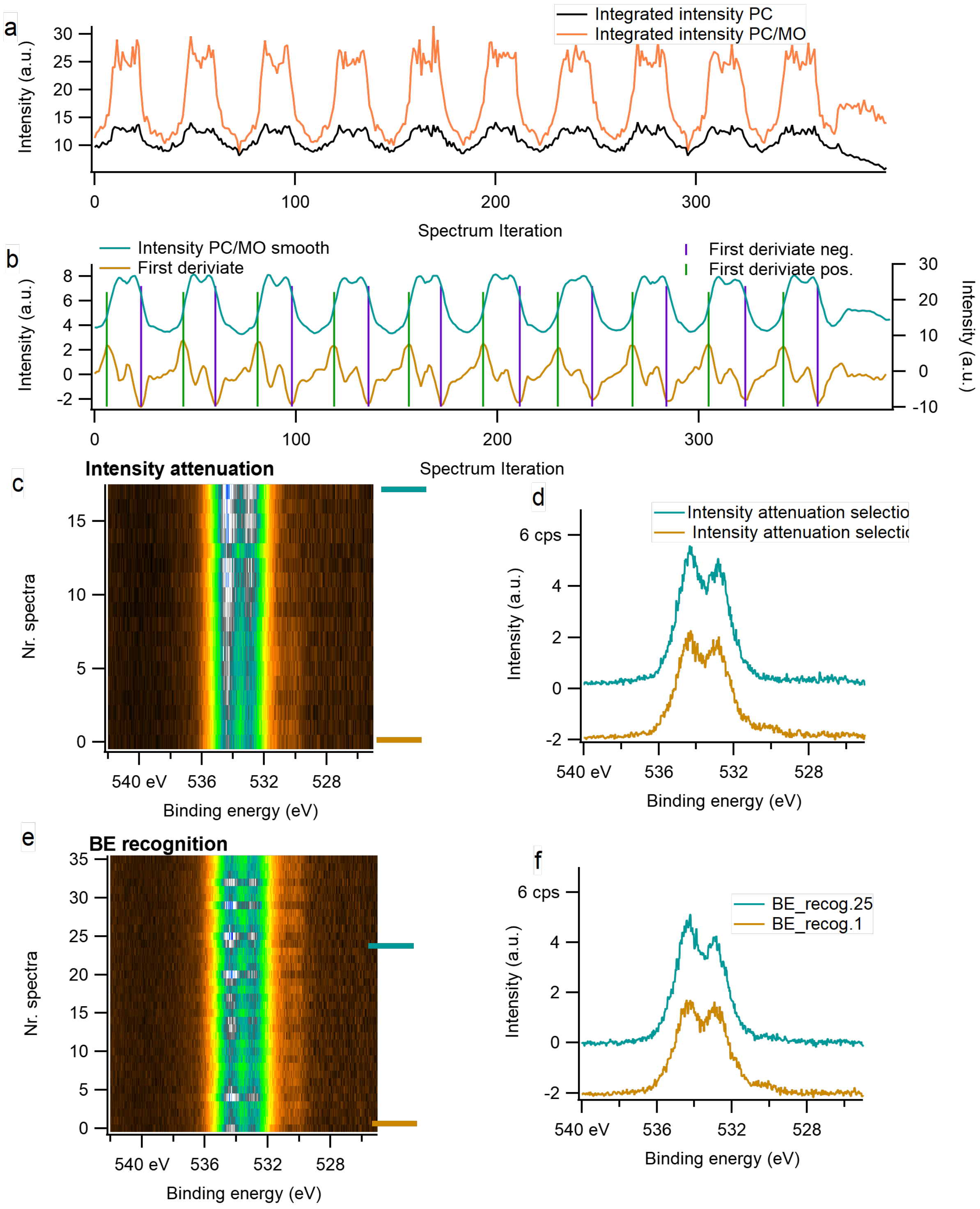

Figure S11: Applying intensity attenuation method for interface recognition in LCO system a) by using the intensity attenuation of the PC/TM oxide peak ratio, and b) after smooth, the peak positions from its first derivative are selected. c) The selection returns a map of all the interface featured spectra and b) two selected interface-featured spectra from the corresponding blue and yellow line positions in c). Slight variation of MO intensity can be seen among the selected spectra.e) Interface feature selection using BE recognition in LCO system, and f) two selected interface-featured spectra from this method.

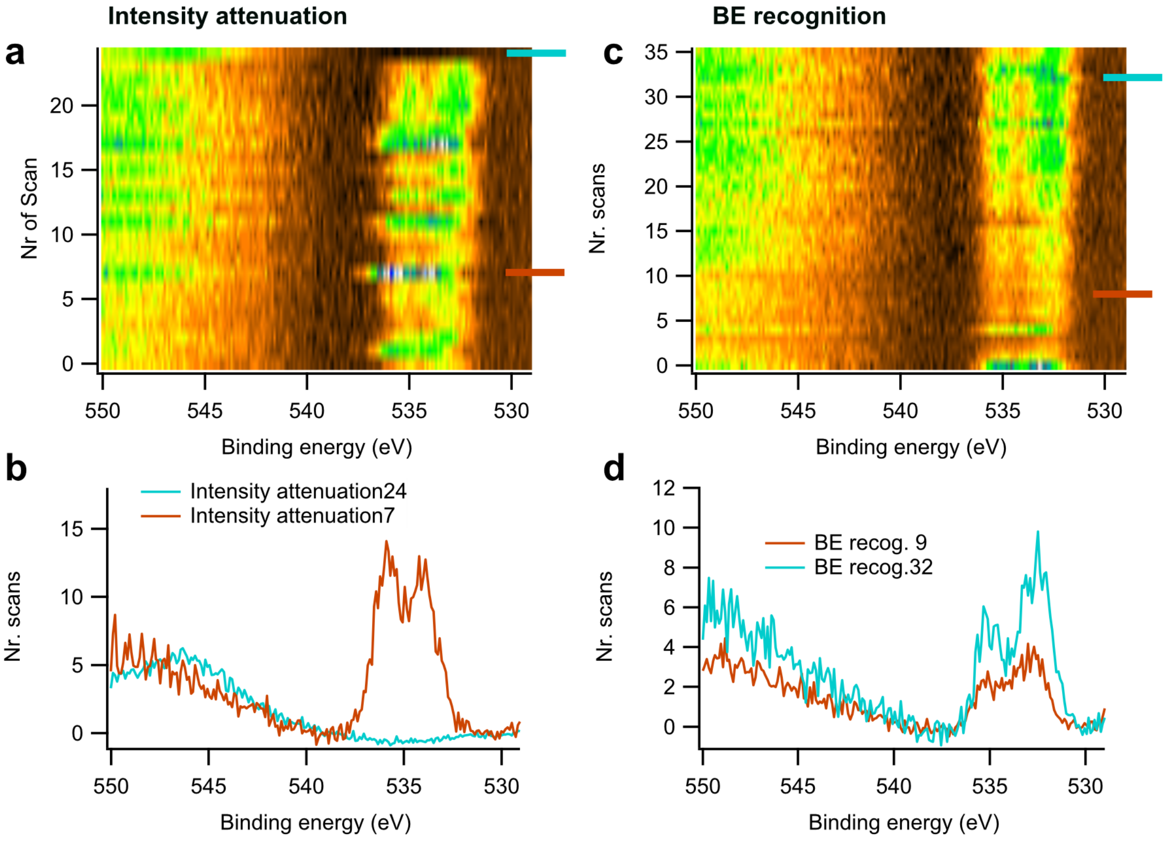

Figure S12: A comparison between selections obtained from BE recognition and Intensity attenuation recognition in the Au system. The intensity-attenuation method produces large variations in spectral features between the two selected spectra, whereas in BE recognition the variation in spectral shape across all selections determined by the BE selection window is much smaller.

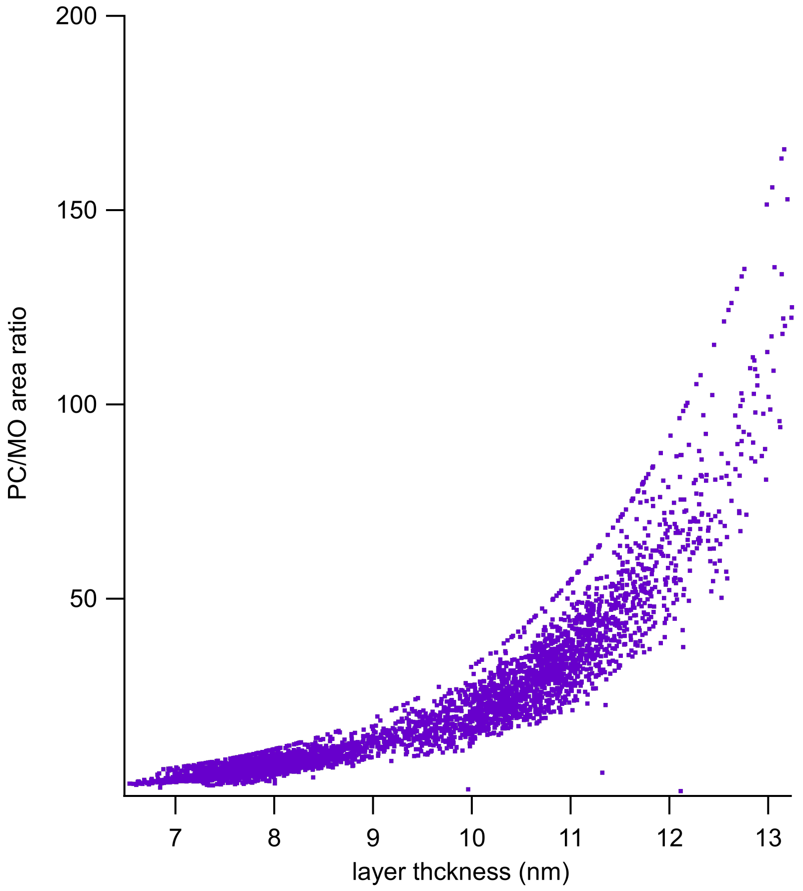

Figure S13: Plot of the PC/MO peak ratio data with respect to the corresponding layer thickness. The exponential corelation reveals $\delta I_{PC/MO}/\delta d$ is not a constant but increases as d increases. In thin liquid regions between 9-10 nm where interface is possibly probed, $\delta I_{PC/MO}/\delta d$ is approx. 10 (a.u./nm), and this ratio quickly increases to 30 when d increases to 11-12 nm. This mathematical relation gives the consequence that the resolution of $I_{PC/MO}$ will decrease as the liquid thickness d increase. Reverse versa, the liquid thickness d has better resolution than peak intensity ratio $I_{PC/MO}$ when they are both plotted against the vertical height.

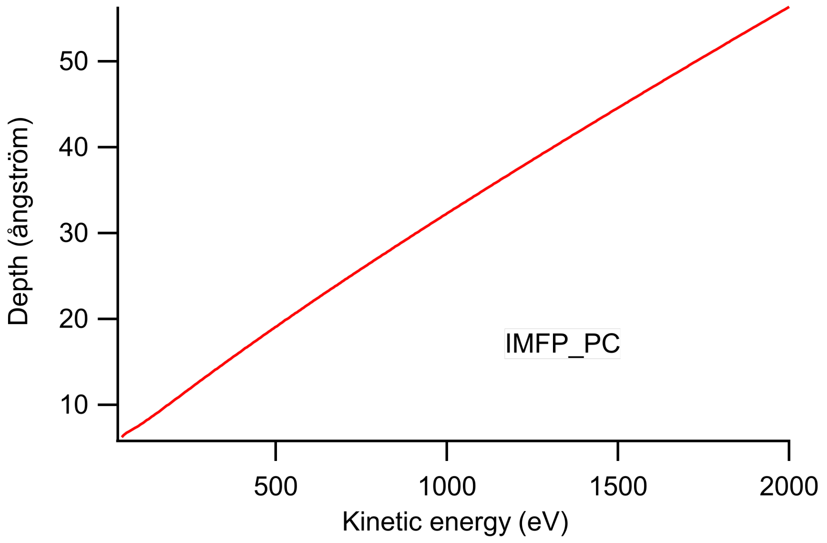

Figure S14: The IMFP of electrons in liquid PC from 50 to 2000 eV kinetic energy, using TPP-2M model.

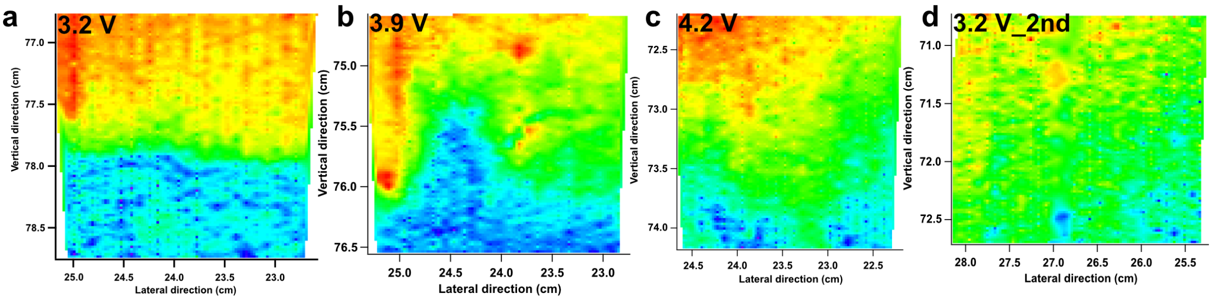

Figure S15: Imaging of surface liquid distribution under different applied potentials.

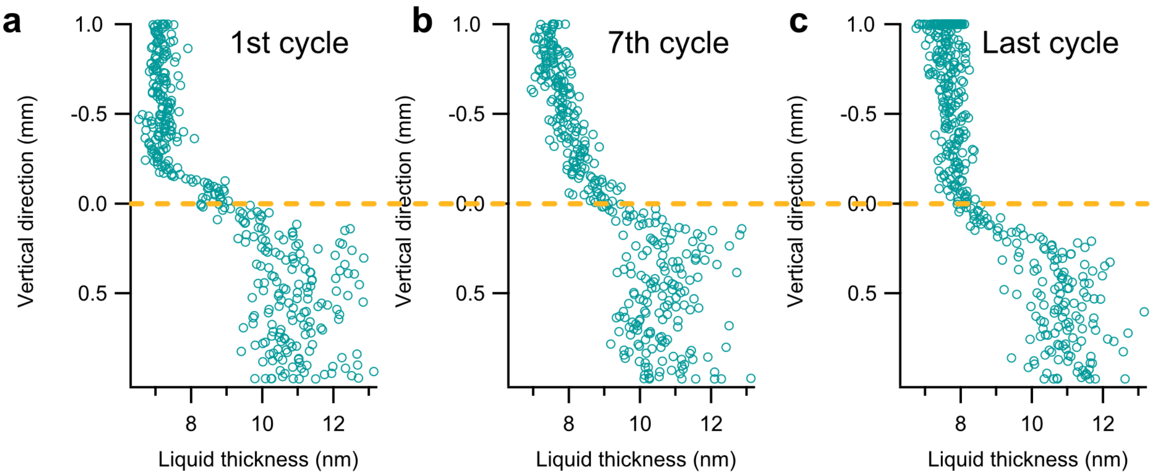

Figure S16: The side views of liquid layer on the LCO sample from a) the first cycle, b) 7th cycle, approximately the middle of the one measurement series, and c) the last cycle of the scanning APXPS measurement at 3.2 V. The thickness difference between the thin and thick liquid region in the probing range becomes smaller from the 1st to the last cycle. The transition position from thin to thick liquid is seen to move downwards compared to that between the first and the last cycle of scanning.

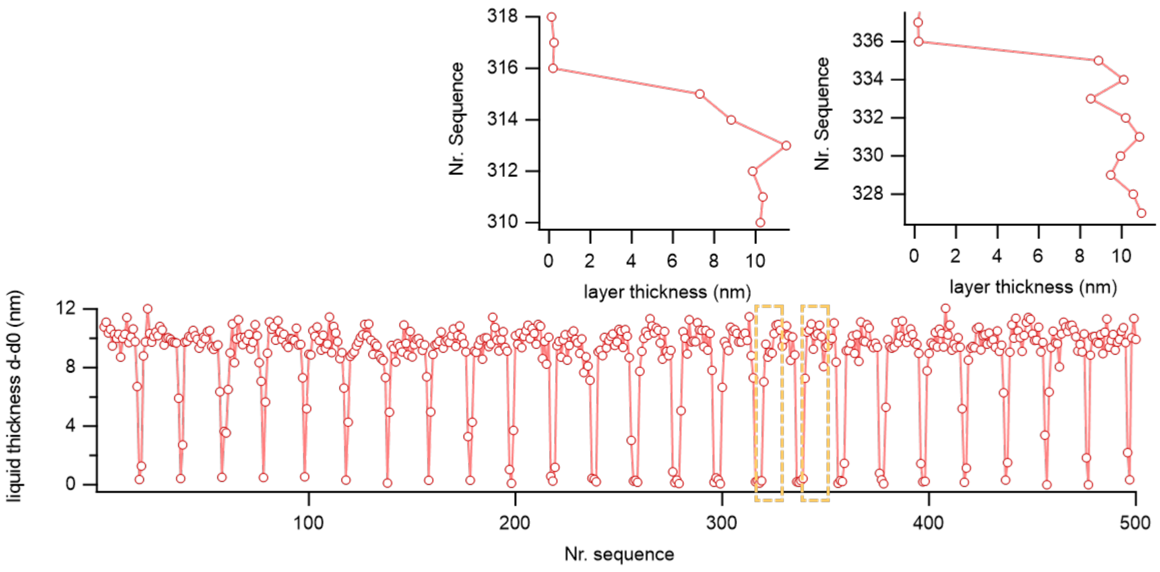

Figure S17: Estimated liquid thickness on Au system from eq.1 in main text.

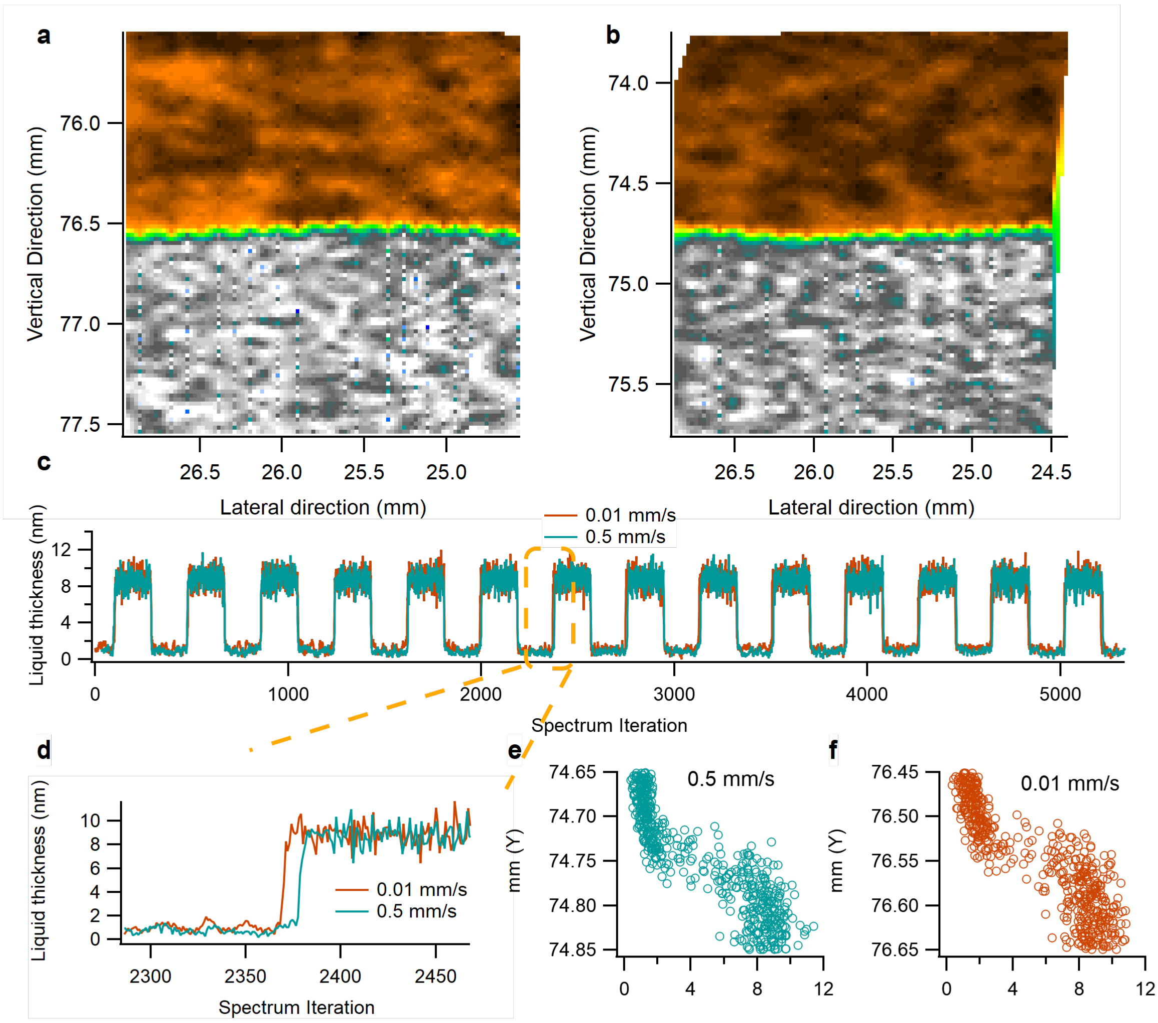

Figure S18: When electrodes were pulled up from the liquid reservoir, the velocity of pulling up was controlled to be 0.1 mm/s for most of the measurements in this paper. To test wether the liquid distribution on the WE surface is influenced by the pulling-up speed, spectro-microscopic imaging of liquid edge on Au WE was applied with pulling up velocity of a) 0.01 mm/s and b) 0.5 mm/s. Both the image from the two measurements shows sharp transitions from dry region to thick liquid region with approximately same width for the intermediate thin liquid. c) The corresponding liquid thickness vs the spectrum iteration with the d) zoom-in of one-half cycle where the liquid thickness growth shows same trend and slope between the two experiments with different pulling-up speeds. Same conclusions are also obtained from the side view of the liquid layer with respect to the vertical position where the shape of the liquid edge shows nearly the same growth curvature.

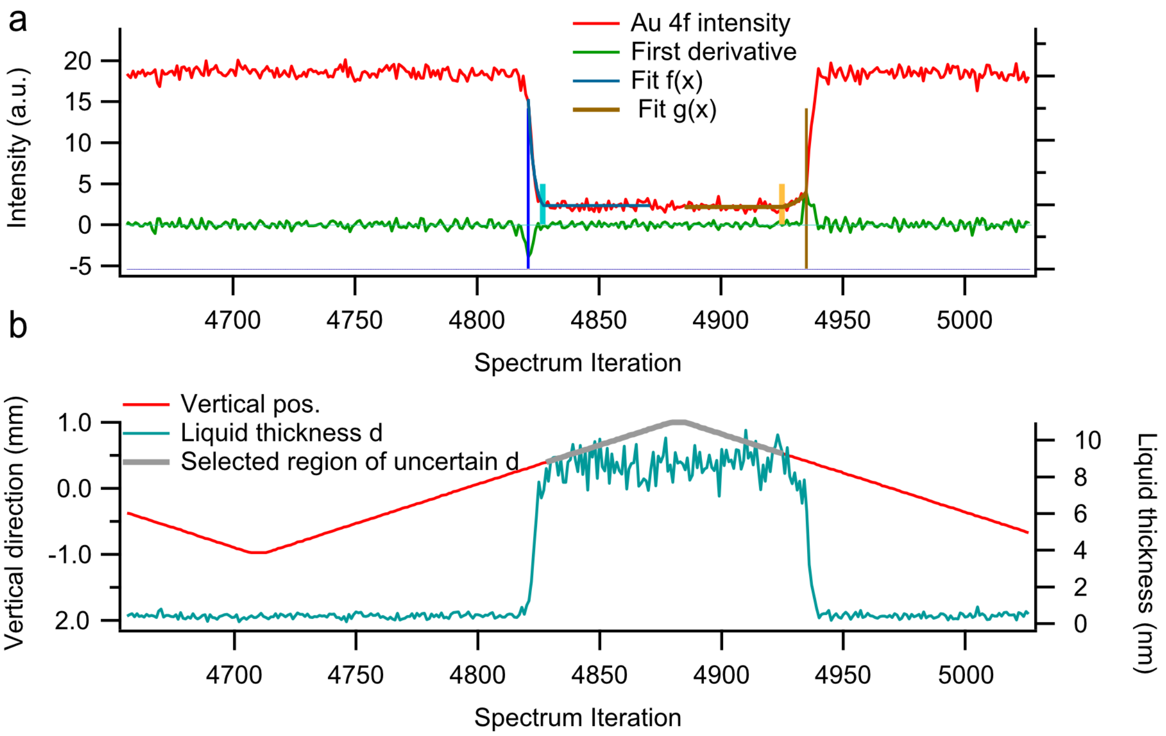

Figure S19: a) The integrated Au 4f peak intensity vs. the spectrum iteration (red). The decay of intensity when scanning from dry to wet region is described by an exponential function combined with a constant as the background level. The curve fit is shown in the blue curve. The fitting region starts from the inflection point of the intensity curve (blue short sticks) determined by the peak positions of the first derivative (green curve) and contains 50 data points onward. The rising of the intensity when scanning upwards from wet to dry region is described by a constant followed by a exponential rise. The curve fitting and the determination of edge position are in the same way as the other side. b) The selected region with no Au intensity is marked gray in the scanning trace (red curve) and their corresponding liquid thicknesses in the blue curve.

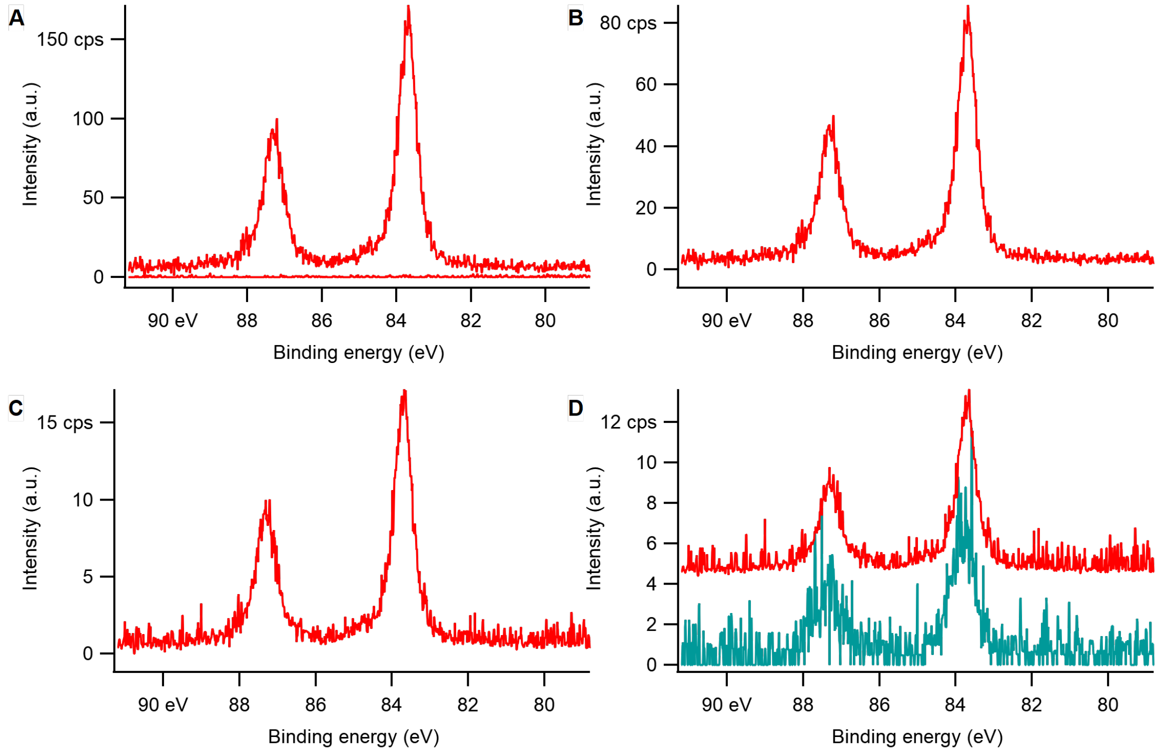

Figure S20: a) Au 4f spectra from probed at thick liquid and dry solid. A sum of the spectra from solid and liquid region with b) half weight from each of them, c) 0.1 weight from the dry solid spectrum and 0.9 from the thick liquid, and d) 0.05 weight from the dry solid spectrum (red spectrum) compared with the selected interface featured spectrum (blue) with the intensity attenuation method.

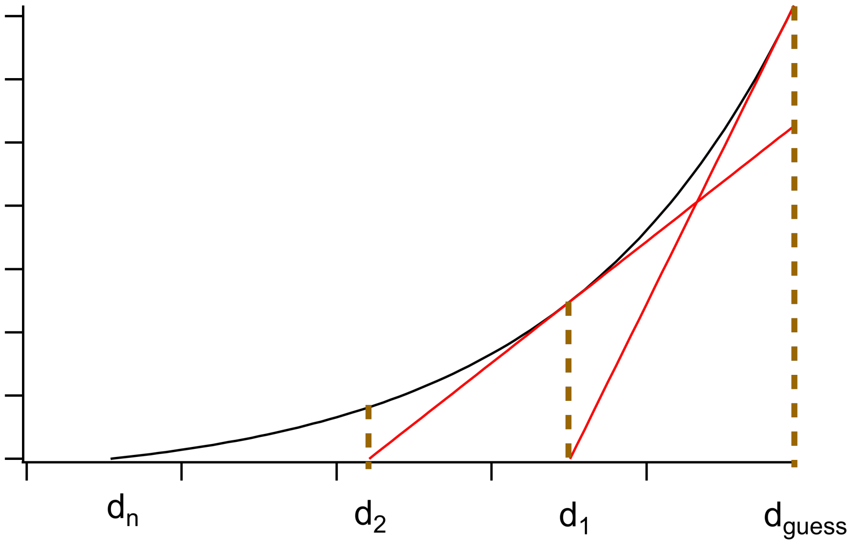

Figure S21: Illustration of finding the root of a function with Newton's method.

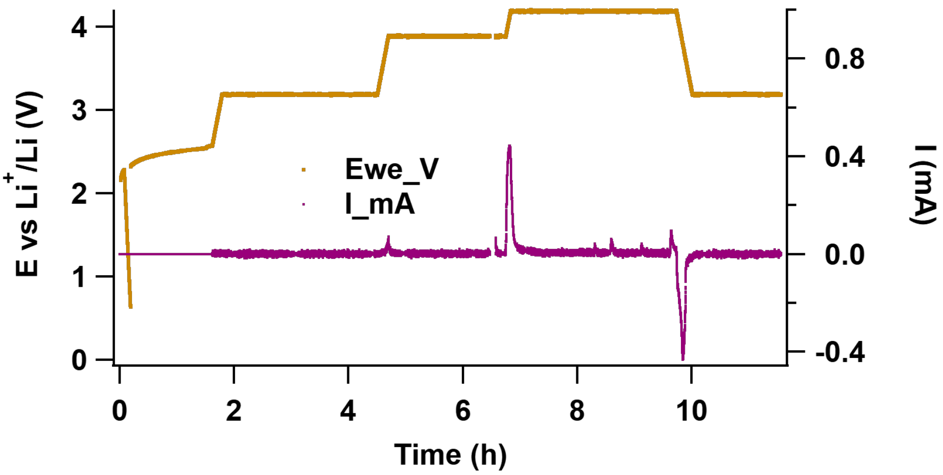

Figure S22: Electrochemical data of LCO thin film during the imaging measurment.

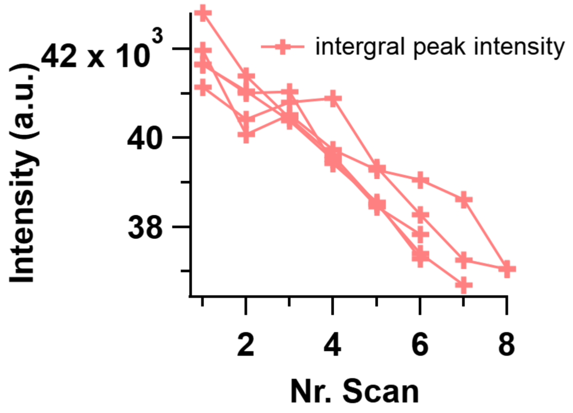

Figure S23: The Signal damping rate of Co 2p under the irradiation.

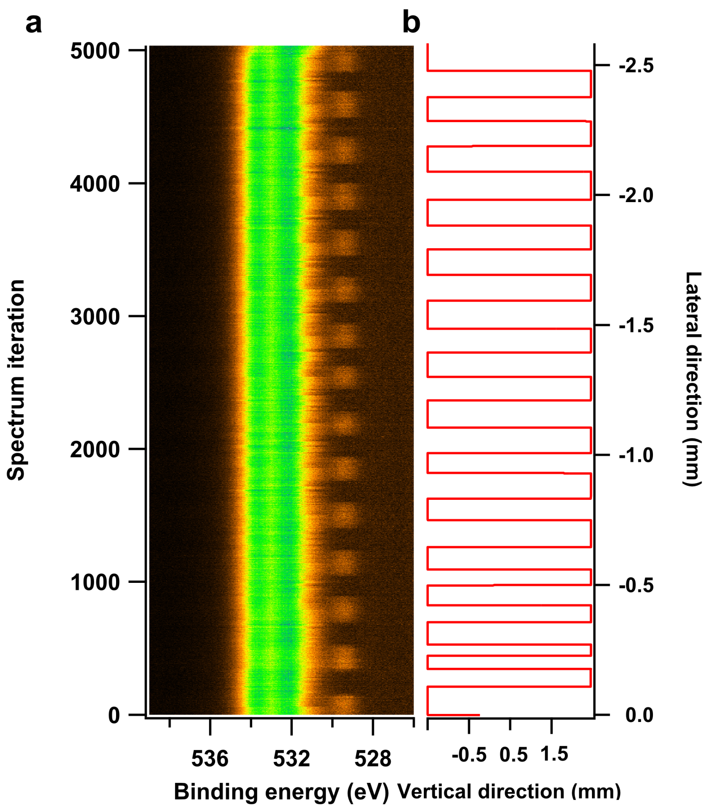

Figure S24: a) The O 1s spectra heatmap from LCO/PC system under an applied voltage of 3.2 V. b) The corresponding scanning trace of each spectrum recorded from the manipulator coordination.

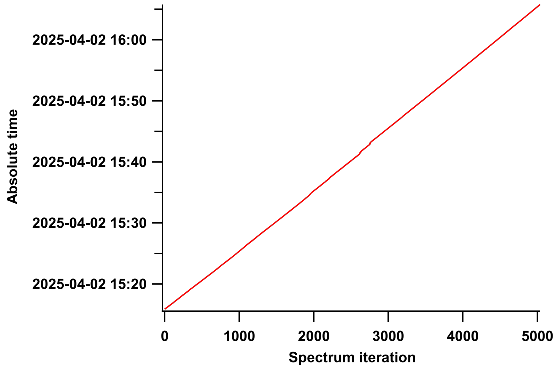

Figure S25: Absolute time of acquisition of individual spectrum during scanning APXPS of 5000 O 1s spectra in LCO system.

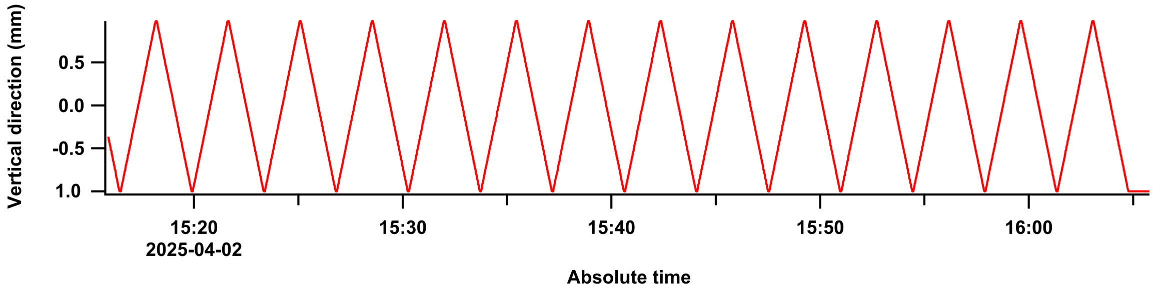

Figure S26: The vertical coordinates of each O 1s spectrum iteration during a scanning APXPS measurement of 5000 iterations.

2 Notes

2.1 XPS spectra intensity calibration

In each series of spectra obtained from scanning APXPS, the background intensity of each spectrum often varies largely as it crosses different regions. This can be from the variation of incoming photon intensities (e.g. injections of beam), de-focusing of beam on some regions due to the spatial un-flatness of the sample, and especially, low inelastic scattering cross-section for liquid phase compared with solid phase. Calibration of intensity is then applied in order for the direct comparison of the peak intensities among spectra in one series, as well as for the later automatic curve-fitting procedure. The intensity calibration is done by normalizing the spectrum intensity to the integrated intensity of its background region (i.e. an energy range applied for the whole series of spectra where no spectral feature is found).

2.2 XPS spectra auto-fitting

The curve fitting for big data set is accomplished by the built-in pseudo-Voigt function in Igor pro software with optimization on multi-component fitting, joint with an automation fitting procedure. Constraint on BE range and peak intensity (only for MO peak on LCO sample) were defined to limit the degree of freedom. An automation curve fitting procedure is employed for fitting multiple spectra in a 2D wave matrix. After the fitting reaches its convergency, the spectrum under calculation is replaced by the next column of data in the matrix for a new fitting process using do-while loops. Simultaneously, the fitting parameters are recorded from where the BEs, intensities etc are gathered. The scripts can be accessed upon request to author.

2.3 Statistical analysis for non-Au-signal recognition

The method was applied on the curve of Au intensity integral vs. the spectrum iteration (Figure S19aa). To identify the data points of which the Au intensity attenuates to its background level in each round, the attenuation of the Au intensity was modelled as an exponential decay combined with a constant as the background level A_{bg}:

$$f(x) = A_{bg} + e^{\frac{x-x_0}{t}} - e^{\frac{k-x_0}{t}}(x \leq k)) \tag{1}$$

$$f(x) = A_{bg}(x \geq k)) \tag{2}$$

where x_0 is an offset parameter and t determines the gradient of the exponential curve. The joint point k of the two functions corresponds to the edge position where the intensity decays to the level of the background and is selected during the function fit (FigureS19 short blue sticks).

A mirror function is applied for the other edge when scanning from thick liquid to the dry region:

$$g(x) = A_{bg1}(x \leq k_1)) \tag{3}$$

$$g(x) = A_{bg1} + e^{\frac{x-x_1}{t_1}} - e^{\frac{k-x_1}{t_1}}(x \geq k_1)) \tag{4}$$

The starting positions of the function fit for both edges are defined by the inflection points of the intensity curve in each cycle (Figure S19 blue and yellow long sticks), and the locations of them were determined by the peak positions of the first derivative (Figure S19a green curve). The regions between the two edge positions given by the value of k and k_1 in each scanning cycle were then marked.
